# The Antiproliferative and Apoptotic Effect of a Novel Synthesized *S*-Triazine Dipeptide Series, and Toxicity Screening in Zebrafish Embryos

**DOI:** 10.3390/molecules26041170

**Published:** 2021-02-22

**Authors:** Azizah M. Malebari, Rakia Abd Alhameed, Zainab Almarhoon, Muhammad Farooq, Mohammad A. M. Wadaan, Anamika Sharma, Beatriz G. de la Torre, Fernando Albericio, Ayman El-Faham

**Affiliations:** 1 Department of Pharmaceutical Chemistry, College of Pharmacy, King Abdulaziz University, Jeddah 21589, Saudi Arabia; amelibary@kau.edu.sa; 2 Department of Chemistry, College of Science, King Saud University, P.O. Box 2455, Riyadh 11451, Saudi Arabia; Roki.ahmed@yahoo.com (R.A.A.); zalmarhoon@ksu.edu.sa (Z.A.); 3 Bio-Products Research Chair, Department of Zoology, College of Science, King Saud University, P.O. Box 2455, Riyadh 11451, Saudi Arabia; Wadaan@ksu.edu.sa; 4 Deanship of Postgraduate Studies and Scientific Research, Almaarefa University, Riyadh 11597, Saudi Arabia; 5 KwaZulu-Natal Research Innovation and Sequencing Platform (KRISP), School of Laboratory Medicine and Medical Sciences, College of Health Sciences, University of KwaZulu-Natal, Durban 4041, South Africa; anamika.aug14@gmail.com (A.S.); garciadelatorreb@ukzn.ac.za (B.G.d.l.T.); 6 Peptide Science Laboratory, School of Chemistry and Physics, University of KwaZulu-Natal, Durban 4001, South Africa; 7 CIBER-BBN (Networking Centre on Bioengineering, Biomaterials and Nanomedicine) and Department of Organic Chemistry, University of Barcelona, 08028 Barcelona, Spain; 8 Institute for Advanced Chemistry of Catalonia (IQAC-CSIC), 08034 Barcelona, Spain; 9 Chemistry Department, Faculty of Science, Alexandria University, P.O. Box 426, Ibrahimia, Alexandria 12321, Egypt

**Keywords:** *s*-triazine, dipeptides, anti-proliferative activity, apoptosis, zebrafish

## Abstract

Several derivatives containing morpholine/piperidine, anilines, and dipeptides as pending moieties were prepared using *s*-triazine as a scaffold. These compounds were evaluated for their anticancer activity against two human breast cancer cell lines (MCF-7 and MDA-MB-231), a colon cancer cell line (HCT-116), and a non-tumorigenic cell line (HEK 293). Tamoxifen was used as a reference. Animal toxicity tests were carried out in zebrafish embryos. Most of these compounds showed a higher activity against breast cancer than colon cancer. Compound **3a**—which contains morpholine, aniline, and glycylglycinate methyl ester—showed a high level of cytotoxicity against MCF-7 cells with IC_50_ values of less than 1 µM. This compound showed a much lower level of toxicity against the non-tumorigenic HEK-293 cell line, and in the in vivo studies using zebrafish embryos. Furthermore, it induced cell cycle arrest at the G2/M phase, and apoptosis in MCF-7 cells. On the basis of our results, **3a** emerges as a potential candidate for further development as a therapeutic drug to treat hormone receptor-positive breast cancer.

## 1. Introduction

Cancer is one of the most threatening diseases for human health. The identification of efficient therapeutic agents to treat this condition is a major objective in medicinal chemistry. Amongst women, breast cancer is considered the most malignant type. Moreover, it is not uncommon for patients to be diagnosed simultaneously with colorectal cancer [1]. New anticancer therapeutic agents should be selective for cancer cells while exerting a lesser or no effect on normal cells [2,3,4]. In order to evaluate the medicinal value of any new compound, it is crucial to determine its biological activity before it can be considered further as a potential drug. Cell-based assays testing the cell viability, cell proliferation, and cytotoxicity are the main and primary steps in the drug discovery process. Among the drugs tested in vitro, only about 2% are approved for clinical trials, but even these may fail in preclinical animal assays [5]. Consequently, testing the safety of newly-synthesized compounds in suitable animal models before clinical trials is an essential step to save time and money. In this regard, zebrafish models have emerged in recent decades for such preclinical testing [6]. Given the reproducibility of the results, zebrafish models are expected to play a key role in speeding up the development of precision medicine [6,7,8,9].

In medicinal chemistry research, cyanuric chloride (trichlorotriazine (TCT)) became a privileged scaffold [10,11] due to its availability and its selective reactivity under a controlled temperature [12,13].

**Figure 1:**
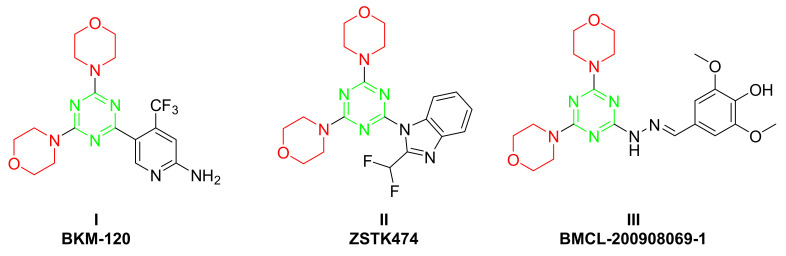
Anticancer s-triazine drugs (**I–III**) in phase III clinical trials.

In this regard, the three Cl atoms can be easily and independently substituted by a variety of nucleophiles in order to render a large number of substituted triazine derivatives. The convenient preparation of these derivatives has allowed their screening against several biological targets. These studies have revealed s-triazine as an outstanding core to build diverse pharmacophores. As such, trisubstituted s-triazine derivatives show numerous biological activities of interest: antimicrobial [14], anticancer [15,16], antitubercular [17], and anti-Human Immunodeficiency Virus anti-HIV [18], as well as inhibitory activity against human monoamine oxidase inhibitors (MAOIs) [19]. Additionally, it is important to highlight several *s*-triazine derivatives with morpholine moieties which show good biological activities, namely BKM-120, **I** [20], ZSTK474, **II** [21], and BMCL-200908069-1, **III** [22], three anticancer agents that have reached phase III clinical trials (Figure 1).

**Figure 2:**
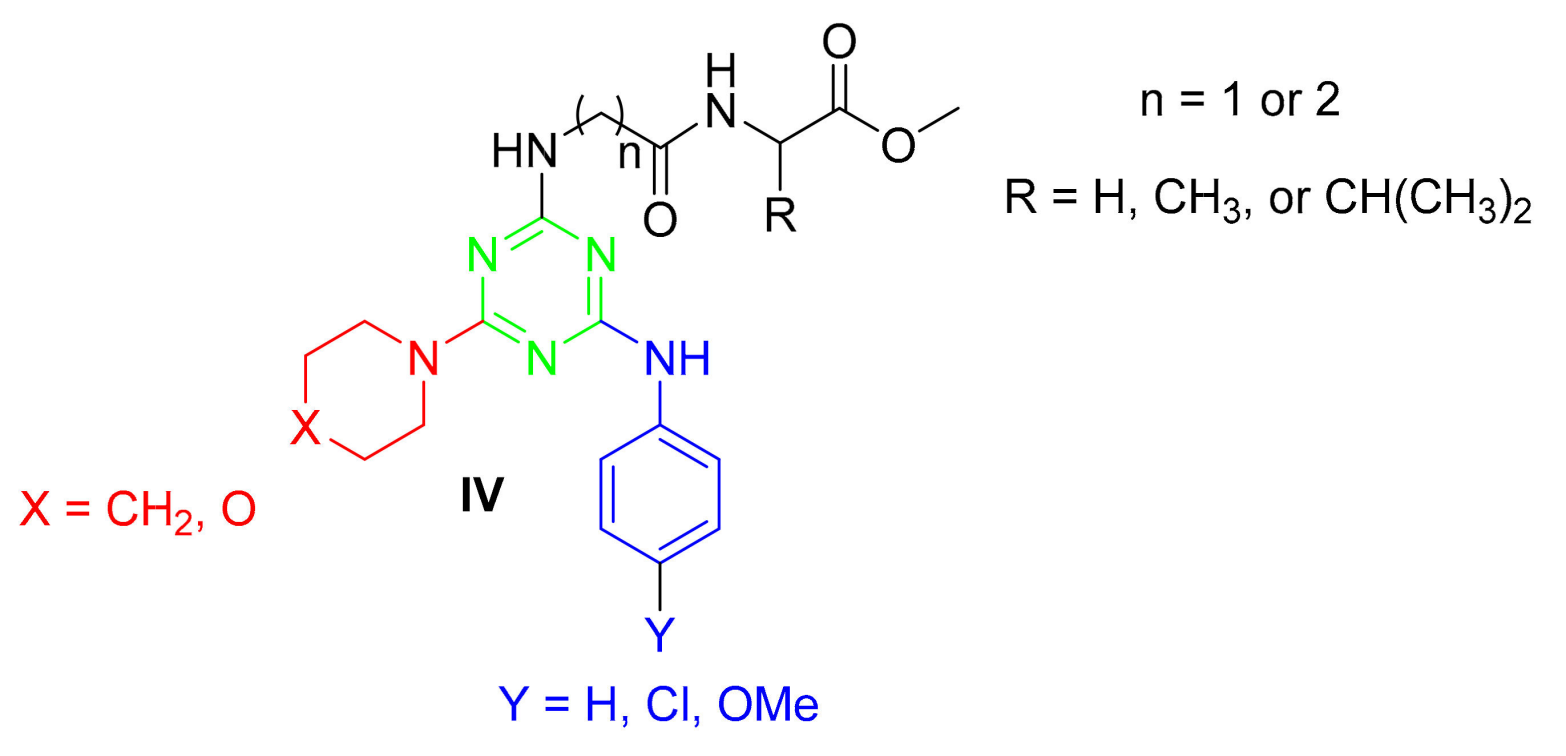
The target *s*-triazinyl dipeptide derivatives.

Given the above data and our ongoing efforts in the design and synthesis of novel antitumor agents using the *s*-triazine scaffold [11,14,15,16,23], here we have focused on the preparation of different *s*-triazine dipeptide (**IV**) derivatives (Figure 2) as modulators of biological activity. The synthesized analogs contain one piperidine/morpholine moiety and one aniline derivative in the other two positions, respectively, which have been previously shown to be important for biological activity. The compounds were evaluated against two human breast cancer cell lines (MCF-7 and MDA-MB-231), one colon cancer cell line (HCT-116), and one non-tumorigenic human embryonic kidney cell line (HEK293). Furthermore, the toxicity of the compounds was also further tested in zebrafish embryos.

## 2. Results and Discussion

### 2.1. Chemistry

The key intermediates—namely 4,6-disubstituted *s*-triazin-2-yl amino acid derivatives **1**—were prepared via a one-pot synthetic strategy using trichloro-s-triazine (TCT) under a controlled temperature, as previously reported by our group [14]. Briefly, TCT was left to react with different nucleophiles at different temperatures, without the isolation of the intermediates. First, the amino acid (Gly or β-Ala) (1 eq.) was added to the TCT using aq. NaHCO_3_ (2.2 eq.) as a base, at 0 °C. After 2 h of reaction, aniline derivatives (1 eq.) were added in the presence of extra aq. NaHCO_3_ (1.2 eq,), and the reaction was left to react overnight at room temperature. Finally, the secondary amine (morpholine or piperidine) (1.1 eq.) was added in the presence of *N*,*N*-diisopropylethylamine (DIEA), and the mixture was refluxed in tetrahydrofuran (THF) for 24 h in order to render **1** in high purity and yield.

The product **1** was reacted with different amino acid methyl ester hydrochloride derivatives (Glycine, Alanine, Valine) **2** using *N*,*N′*-diisopropylcarbodiimide (DIC) and ethyl 2-cyano-2-(hydroxyimino)acetate (OxymaPure) as the coupling method [24] in *N*,*N*-dimethylformamide (DMF) as a solvent and DIEA as a base, in order to give the target products **3a–o** (Scheme 1) in high yield and purity. The spectral data confirmed the structures of the target products (Supplementary Materials, Figures S1–S15).

### 2.2. Biology

#### 2.2.1. Antiproliferative Activity

The antiproliferative activity of the synthesized compounds was evaluated by the 3-(4,5-dimethylthiazol-2-yl)-2,5-diphenyl tetrazolium bromide (MTT) cell viability assay. Two types of breast cancer cell lines—namely MCF-7, which has estrogen and progesterone receptors, and MDA-MB-231, which is triple negative—were used, in addition to a colon cancer cell line (HCT-116). All of the compounds showed an antiproliferative effect against MCF-7, while their activity was slightly lower against MDA-MB-231, and only some of them were active against HCT-116 (Table 1). Overall, the morpholine-containing compounds tended to show greater activity than the piperidine-containing compounds in the MCF-7 cell line. In this regard, it is important to highlight compound **3a**, the activity of which was below µM. As a whole, this compound—which contains morpholino, aniline, and glycinyglycinate as pending moieties of the *s*-triazine ring—was one of the most active in the three cell lines, showing better activity than tamoxifen, which is an estrogen receptor modulator, and has been used as a standard drug in this study [25,26]. Furthermore, **3l**, **3n**, and **3o** also showed low µM activity against the MCF-7 cell line, and good activity against MDA-MB-231 and HCT-116 cells.

**Figure 3:**
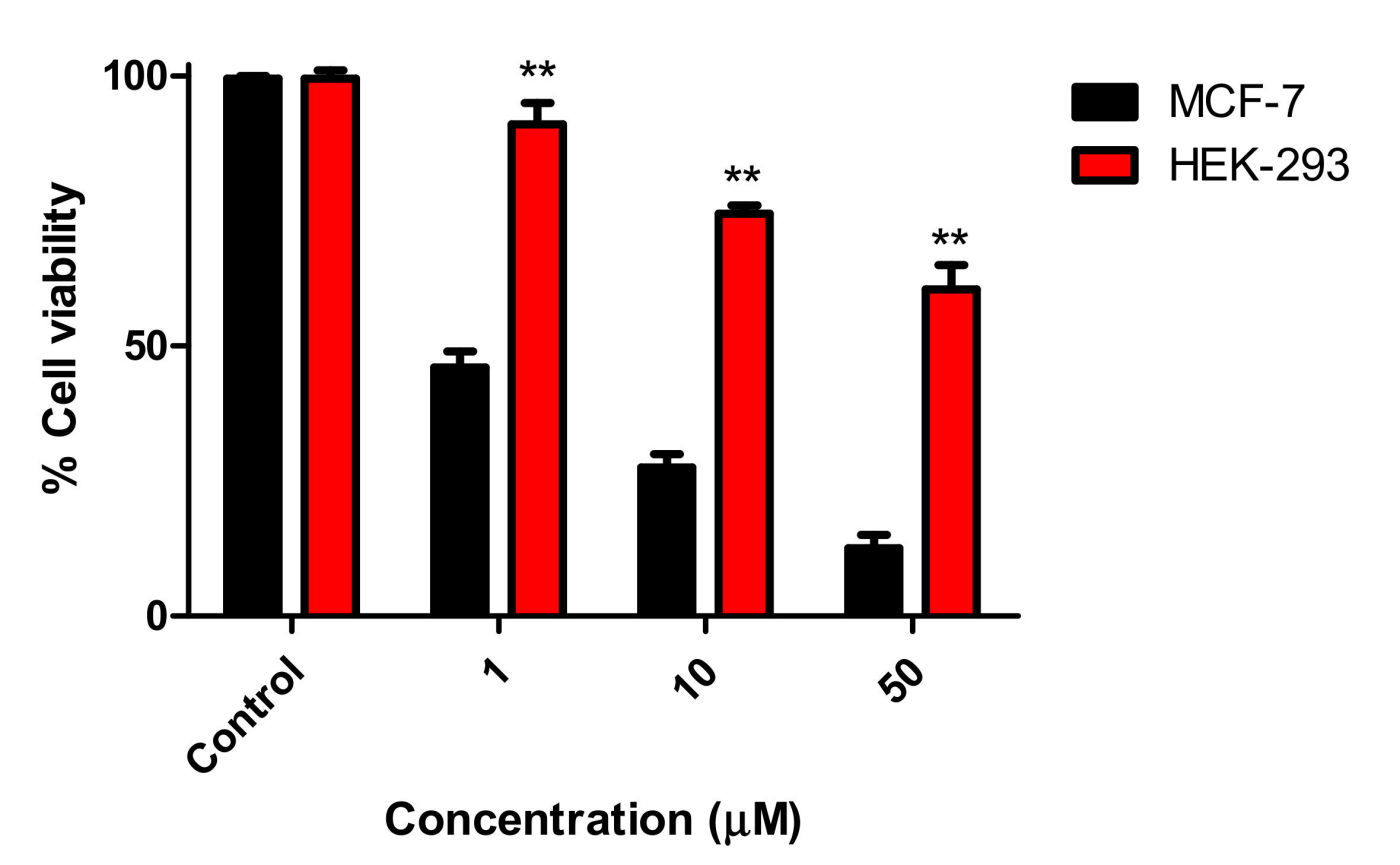
The effect of compound **3a** on the proliferation of breast cancer MCF-7 cells and normal HEK-293 cells. The cells were treated at a concentration of 1, 10 or 50 µM for 72 h. The statistical analysis was performed using a one-way ANOVA-Bonferroni post-hoc test (**, *p* < 0.01).

Additional assays were run with **3a**, the most promising compound. Its toxicity was evaluated using HEK-293 (normal human embryonic kidney), which is a non-tumorigenic cell line. The IC_50_ value of **3a** was > 50 µM, a value that was significantly higher than that observed against the cancer cell lines MCF-7, MDA-MB-231 and HCT-116 (Figure 3). Therefore, this observation demonstrates that **3a** is effectively selective for cancer cells, and causes less toxicity to normal human cells.

#### 2.2.2. Effects on the Cell Cycle Distribution and Apoptosis Assay

**Figure 4:**
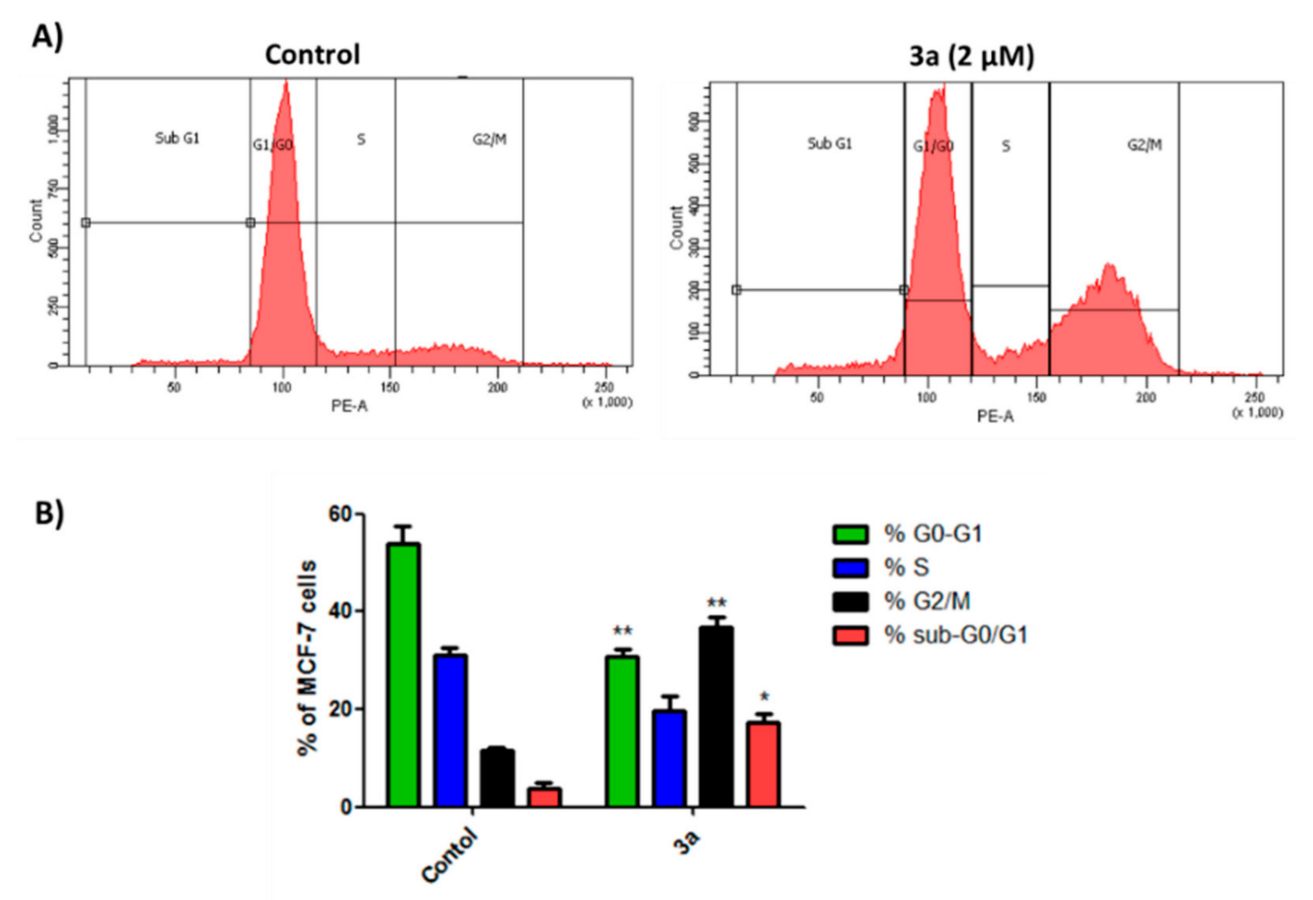
(**A**) Effect of compound **3a** on the cell cycle and apoptosis in breast cancer MCF-7 cells. The cells were treated with either a vehicle (0.1% ethanol (*v*/*v*)) or **3a** (2 µM) for 48 h. The cell cycle analysis was performed on histograms of gated counts per DNA area (FL2-A). (**B**) Quantitative analysis of 4N (G2/M), 2N (G0G1), >2N (S) and <2N (sub-G1). The DNA content was determined with CellQuest software. The values represent the mean ± SEM for three independent experiments. The statistical analysis was performed using a one-way ANOVA-Bonferroni post-hoc test (*, *p* < 0.05; **, *p* < 0.01).

The mechanism underlying the growth-inhibitory effect of **3a** was further examined. The effect of **3a** on cell cycle events in MCF-7 cells, after incubation at 2 µM for 48 h, was analyzed by a DNA flow cytometry assay of propidium iodide (PI) staining (Figure 4A). Compound **3a** induced a statistically significant arrest of MCF-7 cells in the G2/M stage (36.8%) compared to the untreated cell line (11.5%) (Figure 4B). Moreover, the number of cells in the sub-G1 phase increased dramatically, from 3.7% (control) to 17.3%.

**Figure 5:**
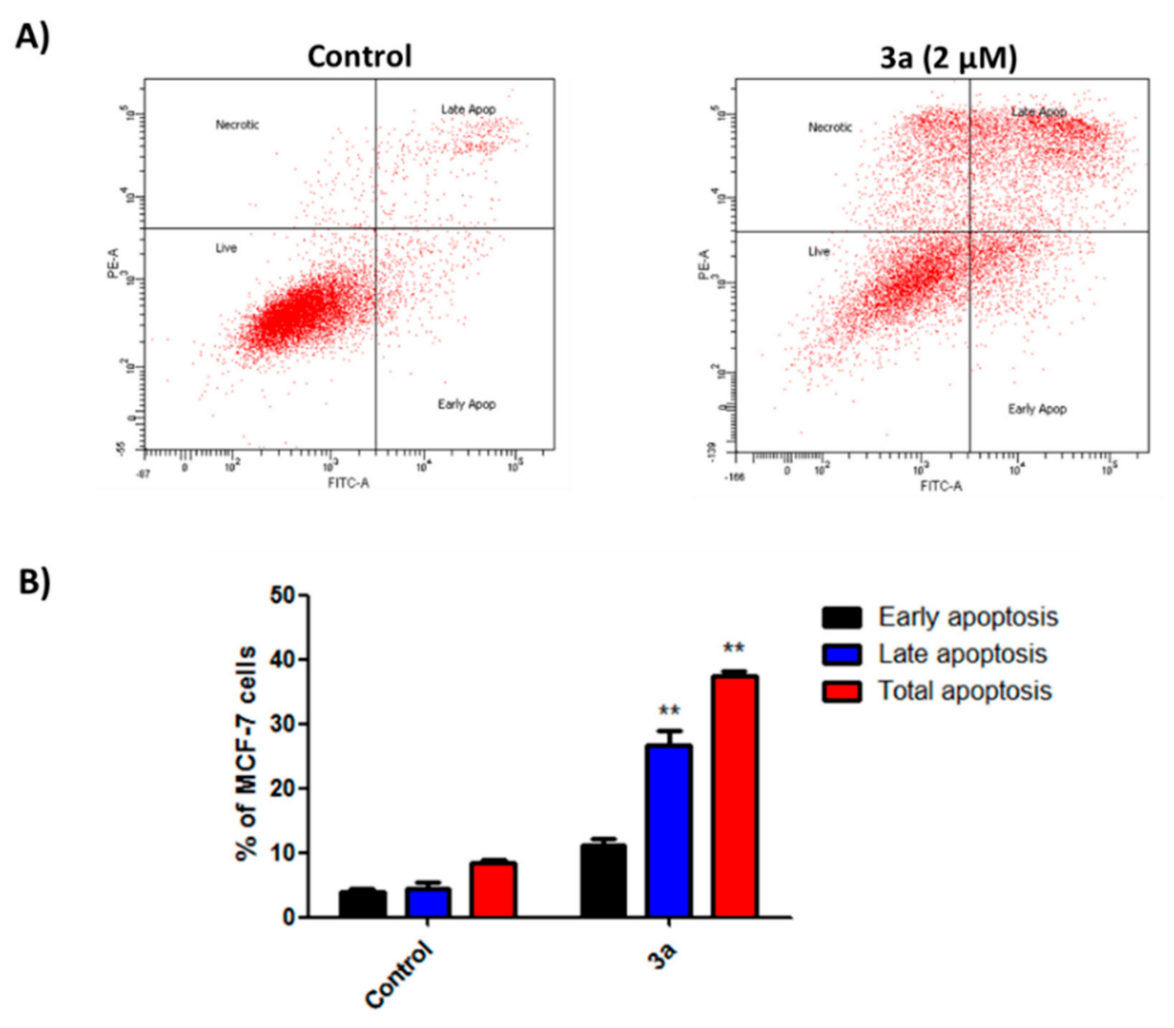
Compound **3a** potently induces apoptosis in breast cancer MCF-7 cells (Annexin V/PI Fluorescence-activated cell sorting (FACS)). (**A**) Flow cytometry analysis of the effect of compound **3a** on MCF-7 cells. The MCF-7 cells were treated with 2 µM of compound **3a** for 48 h, and then collected and processed for analysis. (**B**) The quantitative analysis of the apoptosis. The values represent the mean ± SEM (Standard error of means) for three independent experiments. The statistical analysis was performed using a one-way ANOVA-Bonferroni post-hoc test (**, *p* < 0.01). The four quadrants are identified as follows: lower left (LL), viable; Lower right (LR), early apoptotic; Upper right (UR), late apoptotic; and Upper left (UL), necrotic.

In order to determine the capacity of compound **3a** to induce apoptosis, an Annexin V-FITC/PI dual staining assay was performed on the MCF-7 cells by flow cytometry. At 2 µM, this compound induced both early and late apoptosis in MCF-7 cells when compared to the untreated control cells (Figure 5A). In the control cells, the average proportion of Annexin V-positive cells (total apoptotic cells) increased from 8.3% to 37.4% (Figure 5B). These results revealed that the MCF-7 cells treated with **3a** were arrested at the G2/M phase, and induced mainly cell apoptosis.

#### 2.2.3. In Vivo Evaluation in Zebrafish

The cytotoxicity of compounds **3a–o** was screened in zebrafish embryos. Compounds **3a**, **3b**, **3c**, and **3d** did not show any toxicity in the zebrafish assay and were well tolerated even at the maximum concentration tested (1 mM), where no significant difference in terms of mortality was observed with respect to DMSO as the control (Table 2).

**Figure 6:**
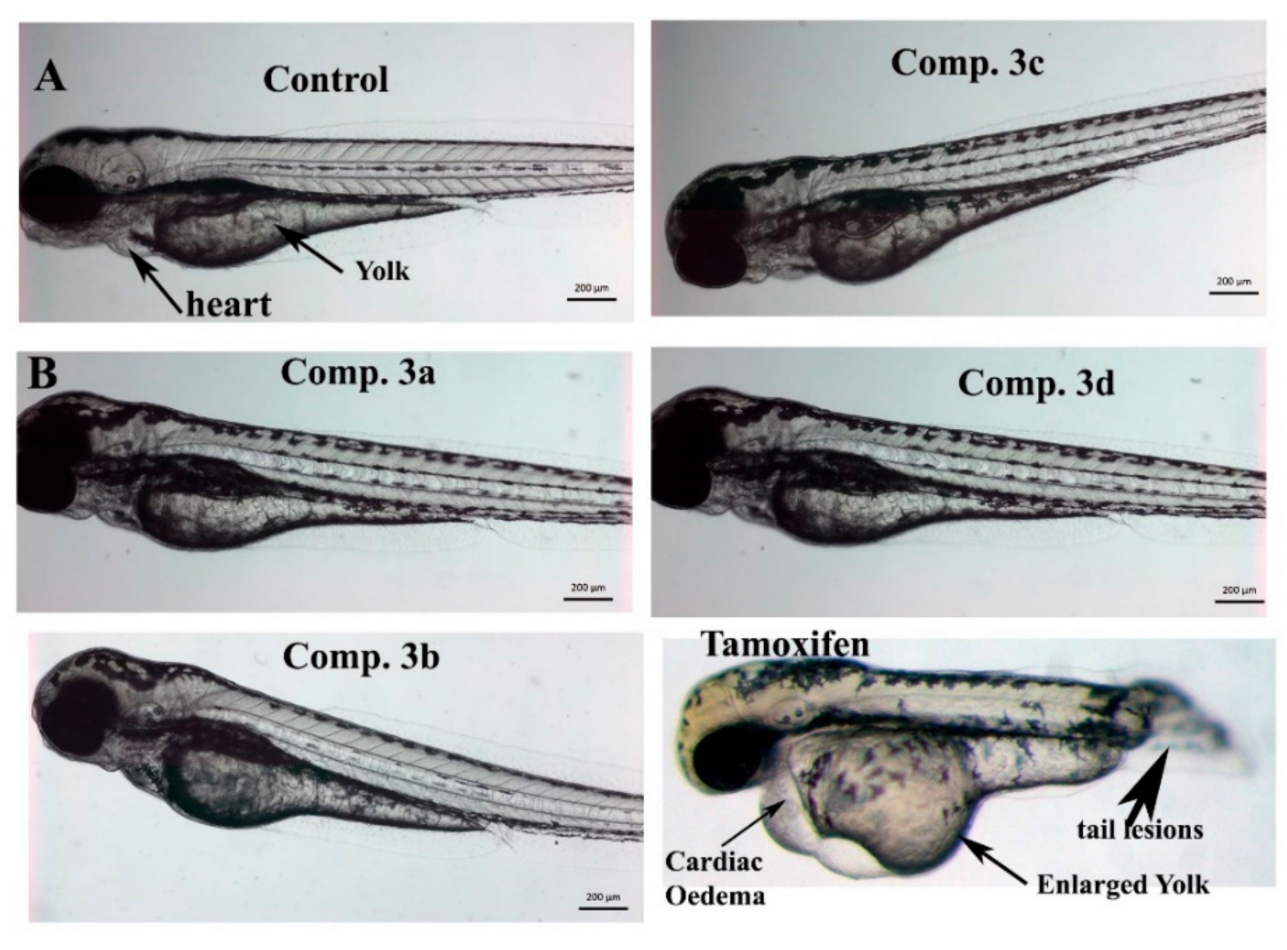
Representative images of the zebrafish embryos 48 hpf (**A**) control or (**B**) treated. Zebrafish embryos shown are treated with 1 mM of compounds **3a, 3b, 3c, 3d,** and 10 µM tamoxifen.

Moreover, the images of the zebrafish embryos at 48 hpf (high power field) did not show any abnormality and/or morphological difference with respect to the non-treated embryos (Figure 6). In contrast, the zebrafish embryos treated with 10 µM tamoxifen showed a severe teratogenic effect. The treated embryos had cardiac edema, craniofacial defects, tail lesions, and a delay in embryonic development. These observations are consistent with the findings in the literature [27,28]. Of note, **3a**, which was the compound that showed the greatest capacity to inhibit MCF-7 cell proliferation, did not show any toxicity.

## 3. Materials and Methods

### 3.1. Materials and Methods

All of the reagents, chemicals and solvents were purchased from commercial suppliers. The reactions were monitored using TLC (silica gel 60-F254 protected aluminum sheets). The melting points were ascertained in open capillary tubes using a Gallenkamp melting point apparatus (Sigma-Aldrich Chemie GmbH, Taufkirchen, Germany), and were uncorrected. The Fourier transform infrared spectroscopy (FTIR) spectra were recorded on a Nicolet 6700 (Thermo Electron Scientific Instruments Corporation, Madison, WI, USA) spectrometer using KBr discs. The ^1^H and ^13^C-NMR spectra were recorded on a Varian-Agilent-NMR 600 MHz spectrometer (Varian, Inc., Palo Altro, CA, USA). The elemental analyses were recorded on a Perkin-Elmer 2400 elemental analyzer (PerkinElmer Inc. Waltham, MA, USA). The ultrasonic bath was purchased from Selecta (Barcelona, Spain). The experiments were performed in a multimode reactor (Monowave 300, Aton Paar GmbH, 1400 W maximum magnetron, Germany). The heat time (5 min, 70 °C, 350 watt) and reaction time (hold time) were 12–15 min at identical wattages and temperatures, with stirring (800 rpm) and cooling until 25 °C.

### 3.2. Chemistry

#### 3.2.1. General Procedure for the Synthesis of the 4,6-Disubstituted s-Triazin-2-yl Amino Acid Derivatives

The 4,6-Disubstituted-s-triazin-2-amino acid derivatives **1** were reported in our previous work [14].

#### 3.2.2. General Method for the Synthesis of the 4,6-Disubstituted s-Triazine Dipeptide Derivatives

A mixture of an acid **1** (1 mmol) and OxymaPure (1 mmol) was dissolved in 5 mL DMF at 0 °C, followed by the dropwise addition of DIC (1.1 mmol) at 0 °C, with pre-activation for 5 min. The amino acid ester hydrochloride **2** (1 mmol) was then added dropwise at the same temperature, in the presence of diisopropylethylamine (DIEA, 1 mmol) as a base. After that, the mixture was stirred at 0 °C for 1 h, and then left overnight under stirring at room temperature. The progress of the reaction was monitored by TLC (CH_2_Cl_2_-hexane; 4:6). Excess water was added when a precipitate formed. The final product was isolated by filtration, and washed with water. If the precipitate did not form, the mixture underwent extraction, washing and drying with ethyl acetate, with 1 N HCl and a saturated solution of Na_2_CO_3_ and NaCl, followed by drying over anhydrous MgSO_4_ in order to obtain the target product.

*Methyl (4-morpholino-6-(phenylamino)-1, 3, 5-triazin-2-yl) glycylglycinate (3a).* A white powder in a 90% yield, mp 195–197 °C. IR (KBr, cm^−1^): 3374, 3315 (NH), 1728, 1652 (CO).^1^H-NMR (DMSO-*d_6_*, ppm): δ 3.60 (7H, brs, CH_2_-O-CH_2,_ COOCH_3_), 3.67 (4H, t *J* = 4.2, 4.8 Hz, CH_2_-N-CH_2_), 3.83-3.91 (4H, m, CH_2_CONHCH_2_CO), 6.89 (1H, t, *J* = 7.2, 7.8, Ar-H), 7.21(2H, t, *J* = 7.8, 8.4 Hz, Ar-H), 7.69 (2H, dd, *J* = 7.2 Hz, Ar-H), 7.12 (1H, s, NH), 8.20 (1H, d, NH), 9.04 (1H, d, NH); ^13^C-NMR (DMSO-*d_6_*, ppm): δ 41.1, 43.7, 44.1, 52.1, 66.5, 119.9, 121.7, 128.7, 140.9, 164.5, 165.1, 166.2, 170.7, 170.9. Analysis calculated for C_18_H_23_N_7_O_4_ (401.43): C, 53.86; H, 5.78; N, 24.43. Found C, 53.99; H, 5.85; N, 24.61.

*Methyl (4-((4-chlorophenyl) amino)-6-morpholino-1, 3, 5-triazin-2-yl) glycylglycinate (3b).* A white powder in a 90% yield, mp 227–229 °C. IR (KBr, cm^−1^): 3375, 3313 (NH), 1724, 1654 (CO). ^1^H-NMR (DMSO-*d_6_*, ppm): δ 3.58 (3H, s, COOCH_3_), 3.60 (4H, brs, CH_2_-N-CH_2_), 3.66 (4H, m, *J* = 4.2 Hz, CH_2_-O-CH_2_), 3.83-3.89 (4H, m, 2CH_2_), 7.19 (1H, s, NH), 7.26 (2H, d, *J* = 8.4 Hz, Ar-H) 7.72 (2H, dd, *J* = 7.2, 8.4 Hz, Ar-H), 8.21 (1H, d, NH), 9.21 (1H, d, NH); ^13^C-NMR (DMSO-*d_6_*, ppm): δ 41.1, 43.7, 44.1, 52.1, 66.4, 121.5, 125.2, 128.6, 140.0,164.3, 165.0, 166.1, 170.7, 170.8. Analysis calculated for C_18_H_22_ClN_7_O_4_ (435.87): C, 49.60; H, 5.09; N, 22.50. Found C, 49.83; H, 5.21; N, 22.77.

*Methyl (4-((4-methoxyphenyl) amino)-6-morpholino-1, 3, 5-triazin-2- yl) glycylglycinate (3c).* A white powder in a 90% yield, mp 209–211 °C. IR (KBr, cm^−1^): 3374, 3310 (NH), 1724, 1654 (CO). ^1^H-NMR (DMSO-*d_6_*_,_ ppm): δ 3.59 (7H, brs, COOCH_3_ & CH_2_-O-CH_2_), 3.65 (4H, brs, CH_2_-N-CH_2_), 3.68 (3 H, s, -OCH_3_), 3.82-3.88 (4H, m, 2CH_2_), 6.80 (2H, d, *J* = 7.8 Hz, Ar-H), 7.55 (2H, t, *J* = 8.4, 13.8 Hz, Ar-H), 8.19 (1H, s, NH), 8.89 (1H, d, NH); ^13^C-NMR (DMSO-*d_6_*_,_ ppm): δ 41.0, 43.7, 44.1, 52.1, 55.6, 66.5, 114.0, 121.6, 134.0, 154.6, 164.3, 165.1, 166.1,170.8, 170.9. Analysis calculated for C_19_H_25_N_7_O_5_ (431.45): C, 52.89; H, 5.84; N, 22.73. Found C, 53.03; H, 5.95; N, 22.98.

*Methyl (4-morpholino-6-(phenylamino)-1, 3, 5-triazin-2-yl) glycylalaninate (3d).* A white powder in a 93% yield, mp 196–198 °C. IR (KBr, cm^−1^): 3400, 3281 (NH), 1740, 1657 (CO).^1^H-NMR (DMSO-*d_6_*_,_ pmm): δ 1.25(3H, d, *J* = 7.2 Hz, NHCH(CH_3_)), 3.57 (3H, s,COOCH_3_), 3.60 (4H, brs, CH_2_-O-CH_2_), 3.66 (4H, m, CH_2_-N-CH_2_), 3.80-3.93 (2H, m, CH_2_), 4.30 (1H, t, *J* = 6.6, 7.2 Hz, CH)*,* 6.89 (1H, t, *J* = 7.2, 7.8, NH), 7.03 (1H, m, Ar-H), 7.21(2H, t, *J* = 7.8, 8.4 Hz, Ar-H), 7.69 (2H, dd, *J* = 8.4, 7.2 Hz, Ar-H), 8.26 (1H, d, NH), 9.02 (1H, d, NH); ^13^C-NMR (DMSO-*d_6_*_,_ ppm): δ 17.6, 43.7, 47.8, 47.9, 52.3, 66.5, 119.9, 121.7,128.7, 140.9, 164.5, 165.0, 166.1, 170.0, 173.5. Analysis calculated for C_19_H_25_N_7_O_4_ (415.45): C, 54.93; H, 6.07; N, 23.60. Found C, 54.76; H, 6.19; N, 23.85.

*Methyl (4-((4-chlorophenyl) amino)-6-morpholino-1, 3, 5-triazin-2-yl) glycylalaninate (3e).* A white powder in a 90% yield, mp 189–191 °C. IR (KBr, cm^−1^): 3408, 3273 (NH), 1745, 1659 (CO). ^1^H-NMR (DMSO-*d_6_*_,_ ppm): δ 1.25 (3H, d, *J* = 6.6 Hz, CH_3_), 3.56-3.60 (7H, m, COOCH_3_ & CH_2_-O-CH_2_), 3.66 (4H, brs, CH_2_-N-CH_2_), 3.79-3.82 (2H, m, CH_2_), 4.29 (1H, q, *J* = 6.6, 7.2 Hz, CH), 7.11 (1H, s, NH), 7.25 (2H, d, *J* = 8.4 Hz, Ar-H), 7.73 (2H, dd, *J* = 7.2, 8.4 Hz, Ar-H), 8.26 (1H, d, NH), 9.19 (1H, d, NH); ^13^C-NMR (DMSO-*d_6_*_,_ ppm): δ 17.6, 43.7, 47.8, 47.9, 52.3, 66.5, 121.4, 125.2, 128. 6, 140.0, 164.4, 165.0, 166.1, 170.0, 173.5. Analysis calculated for C_19_H_24_ClN_7_O_4_ (449.90): C, 50.72; H, 5.38; N, 21.79. Found C, 50.54; H, 5.26; N, 21.99.

*Methyl (4-((4-methoxyphenyl) amino)-6-morpholino-1, 3, 5-triazin-2- yl) glycylalaninate (3f).* A white powder in a 90% yield, mp 191–193 °C. IR (KBr, cm^−1^): 3405, 3286 (NH), 1742, 1660 (CO). ^1^H-NMR (DMSO-*d_6_*_,_ ppm): δ 1.24(3H, d, *J* = 7.2 Hz, CH_3_), 3.59 (7H, brs, CH_2_-O-CH_2_ & COOCH_3_), 3.65 (4H, brs, CH_2_-N-CH_2_), 3.68 (3H, s, -OCH_3_) 3.77-3.80 (2H, m, CH_2_), 4.29 (1H, t, *J* = 6.6 Hz, CH), 6.80 (2H, d, *J* = 8.4 Hz, Ar-H), 7.56 (2H, d, *J* = 7.8 Hz, Ar-H), 8.25 (1H, d, NH), 8.87 (1H, d, NH); ^13^C-NMR (DMSO-*d_6_*_,_ ppm): δ 17.6, 43.7, 47.8, 47.9, 52.3, 55.6, 66.5, 114.0, 121.6, 134.0, 154.6, 164.4, 166.1, 170.1, 173.5. Analysis calculated for C_20_H_27_N_7_O_5_ (445.48): C, 53.92; H, 6.11; N, 22.01. Found C, 54.01; H, 6.30; N, 22.27.

*Methyl (4-((4-chlorophenyl) amino)-6-morpholino-1, 3, 5-triazin-2-yl) glycylvalinate (3g).* A white powder in a 93% yield, mp 173–175 °C. IR (KBr, cm^−1^): 3406, 3367, 3306 (NH), 1727, 1660 (CO). ^1^H-NMR (DMSO-*d_6_*_,_ ppm): δ 0.82 (6H,dd, *J* = 6.6, 10.8 Hz, 2CH_3_), 2.00 (1H, q, *J* = 6.6, 7.2 Hz, CH), 3.58 (4H, brs, CH_2_-O-CH_2_), 3.61 (3H, s, -COOCH_3_), 3.66 (4 H, brs, CH_2_-N-CH_2_), 3.83-3.89 (2H, m, CH_2_), 4.20 (1H, m, CH)_,_ 7.25 (2H, t, *J* = 7.8,8.4 Hz, Ar-H), 7.72 (2H, dd, *J* = 8.4, 7.2 Hz, Ar-H), 8.07 (1H, d, NH), 9.18 (1H, d, NH); ^13^C-NMR (DMSO-*d_6_*_,_ ppm): δ 18.5, 19.4, 30.6, 43.7, 52.1, 57.6, 57.8, 66.5, 121.4, 125.2, 128.6, 140.0, 164.3, 165.0, 166.1, 170.4, 172.5. Analysis calculated for C_21_H_28_ClN_7_O_4_ (477.95): C, 52.77; H, 5.91; N, 20.51. Found C, 52.54; H, 5.86; N, 20.76.

*Methyl (4-(phenylamino)-6-(piperidin-1-yl)-1, 3, 5-triazin-2-yl) glycylglycinate (3h).* A white powder in a 93% yield, mp 92–94 °C. IR (KBr, cm^−1^): 3357 (NH), 1742, 1660 (CO). ^1^H-NMR (DMSO-*d_6_*_,_ ppm): δ 1.46 (4H, brs, 2CH_2_), 1.59 (2H, brs, CH_2_), 3.59 (3H, d, COOCH_3_), 3.67 (4H, brs, CH_2_-N-CH_2_), 3.82-3.90 (4H, m, 2CH_2_), 6.88 (1H, t, *J* = 7.8, 7.2 Hz, Ar-H), 7.02 (1H, s, NH), 7.20 (2H, t, *J = 7.2* Hz, Ar-H), 7.69 (2H, dd, *J* = 7.8, 6.6 Hz, Ar-H), 8.19 (1H, s, NH), 8.95 (1H, d, NH); ^13^C-NMR (DMSO-*d_6_*_,_ ppm): δ 24.8, 25.8, 41.0, 44.0, 44.1, 52.1, 119.8, 121.6, 128.7, 140.9, 164.6, 166.2, 166.6, 170.7, 170.9. Analysis calculated for C_19_H_25_N_7_O_3_ (399.46): C, 57.13; H, 6.31; N, 24.55. Found C, 56.89; H, 6.42; N, 24.78.

*Methyl (4-((4-chlorophenyl) amino)-6-(piperidin-1-yl)-1, 3, 5-triazin-2-yl) glycylglycinate (3i).* A white powder in a 91% yield, mp 172–174 °C. IR (KBr, cm^−1^): 3428, 3278 (NH), 1757, 1658 (CO). ^1^H-NMR (DMSO-*d_6_*_,_ ppm): δ 1.45 (4H, brs, CH_2_), 1.59 (2H, brs, CH_2_), 3.59 (3H, s, COOCH_3_), 3.67 (4 H, s, CH_2_-N-CH_2_), 3.82-3.87 (4H, m, CH_2_), 7.12(1H, s, NH), 7.25 (2H, d, *J* = 8.4 Hz, Ar-H), 7.73 (2H, dd, *J* = 6.6, 7.2 Hz, Ar-H), 8.21 (1H, s, NH), 9.14 (1H, d, NH); ^13^C-NMR (DMSO-*d_6_*_,_ ppm): δ 24.8, 25.9, 41.1, 44.1, 44.1, 52.1, 121.3, 125.0,128.5, 139.9, 164.4, 164.6, 166.1, 166.5, 170.9. Analysis calculated for C_19_H_24_ClN_7_O_3_ (433.90): C, 52.60; H, 5.58; N, 22.60. Found C, 52.83; H, 5.67; N, 22.89.

*Methyl (4-((4-methoxyphenyl) amino)-6-(piperidin-1-yl)-1, 3, 5-triazin-2-yl) glycylglycinate (3j).* White powder in a 88% yield, mp 143–145 °C. IR (KBr, cm^−1^): 3402, 3287 (NH), 1739, 1664 (CO).^1^H-NMR (DMSO-*d_6_*_,_ ppm): δ 1.45 (4H, brs, 2CH_2_), 1.59 (2H, brs, CH_2_), 3.59 (3H, s, COOCH_3_), 3.66 (4H, brs, CH_2_-N-CH_2_), 3.69 (3H, s, OCH_3_), 3.82–3.87 (4 H, m, 2CH_2_), 6.80 (2H, d, *J* = 6.6 Hz, Ar-H), 6.96 (1H, s, NH), 7.57 (2H, t, *J* = 7.2, 12.6 Hz, Ar-H), 8.18 (1H, s, NH), 8.80 (1H, d, NH); ^13^C-NMR (DMSO-*d_6_*_,_ ppm): δ 24.9, 25.8, 41.0, 44.0, 44.2, 52.1, 55.6, 113.9, 121.4, 134.0, 154.5, 164.4, 164.7, 166.2, 170.7, 171.0. Analysis calculated for C_20_H_27_N_7_O_4_ (429.48): C, 55.93; H, 6.34; N, 22.83. Found C, 55.77; H, 6.50; N, 23.04.

*Methyl (4-(phenylamino)-6-(piperidin-1-yl)-1, 3, 5-triazin-2-yl) glycylalaninate (3k).* A white powder in a 90% yield, mp 176–178 °C. IR (KBr, cm^−1^): 3401, 3287 (NH), 1741, 1661 (CO). ^1^H-NMR (DMSO-d_6__,_ ppm): δ 1.25 (3H, d, J = 6.6 Hz, CH_3_), 1.46 (4H, brs, 2CH_2_), 1.60 (2H, brs, CH_2_), 3.58 (3H, s, COOCH_3_), 3.68 (4H, brs, CH_2_-N-CH_2_), 3.83 (2H, m, CH_2_), 4.29 (1H, s, CH)_,_ 6.88 (1H, t, *J* = 7.2, 7.8 Ar-H), 7.20 (2H, t, *J* = 7.8 Hz, Ar-H), 7.69 (2H, dd, *J* = 7.2, 7.8 Hz, Ar-H), 6.93 (1H, s, NH), 8.25 (1H, d, NH), 8.93 (1H, m, NH). ^13^C-NMR (DMSO-*d_6_*_,_ ppm): δ 17.6, 24.8, 25.9, 44.0, 47.9, 52.3, 119.8, 121.6, 128.7, 141.1, 164.5, 164.6, 166.2, 170.1, 173.5. Analysis calculated for C_20_H_27_N_7_O_3_ (413.48): C, 58.10; H, 6.58; N, 23.71. Found C, 58.33; H, 6.64; N, 23.97.

*Methyl (4-((4-chlorophenyl) amino)-6-(piperidin-1-yl)-1, 3, 5-triazin-2-yl) glycylalaninate (3l).* A white powder in a 93% yield, mp 192–194 °C. IR (KBr, cm^−1^): 3400, 3291 (NH), 1745, 1659 (CO). ^1^H-NMR (DMSO-*d_6_*_,_ ppm): δ 1.24 (3H, t, *J* = 7.8, 8.4 Hz, CH_3_), 1.45 (4H, brs, 2CH_2_), 1.59 (2H, brs, CH_2_), 3.58 (3H, s, COOCH_3_), 3.67 (4H, s, CH_2_-N-CH_2_), 3.77-3.89 (2H, m, CH_2_), 4.28 (1H, q, *J* = 6.6,7.2 Hz, CH), 7.02 (1H, s, NH), 7.25 (2H, d, *J=* 7.2 Hz, Ar-H), 7.74 (2H, dd, *J* = 7.2, 8.4 Hz, Ar-H), 8.27 (1H, d, NH), 9.12 (1H, d, NH); ^13^C-NMR (DMSO-*d_6_*_,_ ppm): δ 17.6, 24.8, 25.9, 44.0, 47.9, 47.9, 52.3, 121.3, 125.0, 128.5, 140.2, 164.4, 164.5, 166.1, 170.1, 173.4. Analysis calculated for C_20_H_26_ClN_7_O_3_ (447.92): C, 53.63; H, 5.85; N, 21.89. Found C, 53.85; H, 5.93; N, 21.65.

*Methyl (3-((4-((4-chlorophenyl) amino)-6-morpholino-1, 3, 5-triazin-2-yl) amino) propanoyl) glycinate (3m).* A white powder in a 94% yield, mp 146–148 °C. IR (KBr, cm^−1^): 3368 (NH), 1726 & 1678 (CO). ^1^H-NMR (DMSO-*d_6_*_,_ ppm): δ 2.42 (2H, q, *J* = 7.2, 7.8 Hz, CH_2_), 3.46 (2H, q, *J* = 6.6, 7.2 Hz, CH_2_), 3.59 (4 H, m, CH_2_-O-CH_2_), 3.61 (3H, s, -COOCH_3_), 3.66 (4H, m, CH_2_-N-CH_2_), 3.82 (2H, d, *J* = 6.0 Hz, CH_2_), 6.88 (1H, d, NH), 7.25 (2H, t, *J* = 9.0, 9.6 Hz, Ar-H), 7.75 (2H, d, *J* = 7.8 Hz, Ar-H), 8.33 (1H, s, NH), 9.16 (1H, d, NH); ^13^C-NMR (DMSO-*d_6_*_,_ ppm): δ 35.4, 37.1, 40.9, 43.8, 52.2, 66.7, 121.4, 125.4, 128.8, 140.2, 164.2, 165.1, 166.1, 171.0, 171.9. Analysis calculated for C_19_H_24_ClN_7_O_4_ (449.90): C, 50.72; H, 5.38; N, 21.79. Found C, 50.93; H, 5.43; N, 21.96.

*Methyl (3-((4-((4-chlorophenyl) amino)-6-morpholino-1, 3, 5-triazin-2-yl) amino) propanoyl) valinate (3n).* A white powder in a 92% yield, mp 171–173 °C. IR (KBr, cm^−1^): 3432, 3353, 3194, (NH), 1730, 1679 (CO). ^1^H-NMR (DMSO-*d_6_*_,_ ppm): δ 0.84 (6H, d, *J* = 7.2 Hz, CH_3_CHCH_3_), 1.98 (1H, d, *J* = 6.0 Hz, CH(CH_3_)_2_), 2.47(2H, t, *J* = 5.4, 9.0 Hz, triazine CH_2_CH_2_CONH), 3.46 (2H, t, *J* = 5.4, 6.6 Hz, triazine NHCH_2_), 3.59 (3H, s, COOCH_3_), 3.61–3.68 (8H, m, 4CH_2_), 4.16 (1H, d, *J* = 4.8 Hz, CH), 6.86 (1H, s, NH), 7.24 (2H, d, *J* = 6.6 Hz, Ar-H), 7.75 (2H, s, Ar-H), 8.17 (1H, m, NH), 9.17 (1H, d, NH); ^13^C-NMR (DMSO-*d_6_*_,_ ppm): δ 18.7, 19.4, 30.3, 35.4, 37.2, 43.7, 52.1, 57.8, 66.5, 121.3, 125.2, 128.6, 140.0, 164.4, 165.8, 166.0, 171.6, 172.6. Analysis calculated for C_22_H_30_ClN_7_O_4_ (491.98): C, 53.71; H, 6.15; N, 19.93. Found C, 53.95; H, 6.32; N, 19.77.

*Methyl (3-((4-((4-chlorophenyl) amino)-6-(piperidin-1-yl)-1, 3, 5-triazin-2- yl) amino) propanoyl) valinate (3o).* A white powder in a 91% yield, mp 178–180 °C. IR (KBr, cm^−1^): 3436, 3353, 3194 (NH), 1730 & 1678 (CO). ^1^H-NMR (DMSO-*d_6_*_,_ ppm): δ 0.85(6H, d, *J* = 7.2 Hz, 2CH_3_), 1.45(4H, brs, 2CH_2_), 1.59 (2H, brs, CH_2_), 1.96-2.00 (1H, m, CH), 2.45 (2H, dd, *J* = 7.2, 6.6 Hz, CH_2_), 3.45 (2H, s, CH_2_), 3.61 (3H, s, COOCH_3_), 3.67 (4 H, d, CH_2_-N-CH_2_), 4.16 (1H, t, *J* = 6.6, 7.2 Hz, CH), 6.76 (1H, d, NH), 7.24 (2H, d, *J* = 7.8 Hz, Ar-H), 7.76 (2H, s, Ar-H), 8.17 (1H, t, NH), 9.08 (1H, d, NH); ^13^C-NMR (DMSO-*d_6_*_,_ ppm): δ 18.8, 19.4, 24.8, 25.9, 30.3, 35.4, 37.2, 44.0, 52.1, 57.8, 121.2, 124.9,128.6, 140.2, 164.4, 164.6, 166.2, 171.5, 172.6. Analysis calculated for C_23_H_32_ClN_7_O_3_ (490.01): C, 56.38; H, 6.58; N, 20.01. Found C, 56.14; H, 6.69; N, 20.28.

### 3.3. Biology

#### 3.3.1. MTT Cell Proliferation Assays

The target products **3a–o** were evaluated for their antiproliferative activity using the MTT viability assay of the MCF-7, MDA-MB-231 and HCT-116 cell lines (in triplicate) on at least three independent occasions for the determination of the reported mean values.

#### 3.3.2. Cell Culture

The three human tumor cell lines MCF-7, MDA-MB-231 and HCT-116 were cultured in Dulbecco’s Modified Eagle’s Medium (DMEM) with 10% fetal bovine serum, 2 mM L-glutamine and 100 µg/mL penicillin/streptomycin. The cells were maintained at 37 °C in 5% CO_2_ in a humidified incubator. All of the cells were sub-cultured 3 times/week by trypsinization using TrypLE Express (1×).

#### 3.3.3. Cell Viability Assay

In order to calculate the relative IC_50_ values, the antiproliferative effect of compounds **3a–o** was tested in three cancer cell lines (MCF-7, MDA-MB-231, HCT-116) and normal epithelial embryonic kidney cells (HEK-293) using the MTT viability assay. The cells were seeded in 96-well plates at a density of 10 × 10^3^ cells/mL, in a total volume of 200 µL per well. In total, 0.1% DMSO was used as a vehicle control. After 24 h, the cells were treated with 2 µL of the test compound, which had been pre-prepared as a stock solution to furnish the concentration range of the study (0.01–100 µM), and re-incubated for 72 h. The culture medium was then removed, and the cells were washed with phosphate-buffered saline (PBS), and 100 µL MTT was added (for a final concentration of 5 mg/mL MTT). The cells were incubated for 4 h in darkness at 37 °C. Next, 200 µL DMSO was added to each well, and the cells were maintained at room temperature in darkness for 20 min. The absorbance was detected with a microplate reader at 570 nm. The results were expressed as a percentage of viability relative to the vehicle control (100%). The dose-response curves were plotted, and the IC_50_ values were obtained using Prism software (GraphPad Software, Inc., La Jolla, CA, USA). All of the experiments were repeated in three independent experiments.

#### 3.3.4. Cell Cycle Analysis

The MCF-7 cells were treated with compound **3a** (2 µM) and incubated for 48 h. After trypsinization, the cells were collected and centrifuged at 800× *g* for 15 min. They were then fixed in ice-cold 70% ethanol overnight at −20 °C. The fixed cells were centrifuged at 800× *g* for 15 min and stained with 50 μg/mL PI, containing 50 μg/mL DNase-free RNase A, at 37 °C for 30 min. The DNA content of the cells (10,000 cells) was analyzed by flow cytometer at 488 nm, using a FACSCalibur flow cytometer (BD Biosciences, San Jose, CA, USA).

#### 3.3.5. Annexin V/PI Apoptotic Assay

Flow cytometry using Annexin V and propidium iodide (PI) was performed in order to detect apoptotic cell death. MCF-7 cells were seeded at a density of 1 × 10^5^ cells/well, treated with the vehicle (0.1% (*v*/*v*) EtOH) or compound **3a** (2 µM), and incubated for 48 h. The cells were then harvested and washed twice in 1X binding buffer, and incubated in the dark for 30 min on ice in Annexin V-containing binding buffer [1:100]. They were then re-suspended in PI-containing binding buffer [1:1000]. The samples were analyzed immediately using a BD Accuri flow cytometer, and GraphPad Prism software version 5.01 was used to analyze the data. Four populations were identified during the assay: Annexin V-negative and PI-negative (Q4, healthy cells), Annexin V-positive and PI-negative (Q3, early apoptosis), Annexin V-positive and PI-positive (Q2, late apoptosis), and Annexin V-negative and PI-positive (Q1, necrosis).

#### 3.3.6. Zebrafish Embryo Treatments

The zebrafish embryos were obtained by natural pairwise breeding, and were treated essentially as described previously [29].

The compounds were dissolved in dimethylsulfoxide (DMSO) in order to prepare a stock concentration of 10 mM. The embryos treated with 0.10% DMSO (*v*/*v*) served as the negative controls.

#### 3.3.7. Imaging and Microscopy

A Zeiss Observer D1 inverted microscope using Zeiss ZEN software was used to capture the images of the live zebrafish embryos.

Wild-type zebrafish were maintained at 28.5 °C in a 10 h dark and 14 h light period in a circulating water system provided by Pentair (www.pentair.com). The fish were kept following the national and international guidelines for the care and use of laboratory animals. The fertilized eggs were obtained by the natural pairwise breeding of adult fish. The approval of the Ethics Committee for the use and care of laboratory animals was not needed, as zebrafish larvae less than 5 days post fertilization were used [30].

A stock solution of 50 mM was prepared by dissolving the compounds in dimethyl sulfoxide (DMSO). At least 30 fertilized eggs were sorted and placed in 60-mm sterile glass Petri dishes. A serial dilution of the compounds was prepared by adding the required amount of the compound directly into 5 mL water, and the zebrafish embryos were then exposed to serial dilutions (0.5 to 1000 µM) of each compound overnight. The number of dead and living embryos were counted after the overnight exposure.

## 4. Conclusions

All the synthesized *s*-triazine dipeptides derivatives showed some biological activity, compound **3a** (Methyl (4-morpholino-6-(phenylamino)-1, 3, 5-triazin-2-yl) glycylglycinate) was identified as a novel potent anticancer agent. Compounds **3a** showed excellent activity against the MCF-7and MDA-MB-231 cancer cell lines, with IC_50_ values of 0.82 and 9.36 µM, respectively, compared to the reference drug tamoxifen (IC_50_ = 5.12 and 15.01 µM in MCF-7 and MDA-MB-231 cells, respectively). This can be an indication of certain selectivity in front of those cells that overexpress estrogen and progesterone receptors. In addition, **3a** showed some selectivity for the cancer cells, since the IC_50_ against non-tumorigenic HEK-293 cells was higher than 50 µM, which represents almost two orders of magnitude of difference. The in vivo studies using zebrafish embryos confirmed the low toxicity of compound **3a.** In this regard, up to a concentration of 1 mM, this compound showed the same mortality ratio as the control. Furthermore, the images of the treated embryos did not indicate any embryonic abnormality or morphological difference compared with the untreated embryos.

Finally, the study of the MCF-7 cell cycle revealed that compound **3a** arrests the cycle in the G2/M phase, which is associated with enhanced apoptosis. This finding was demonstrated in the Annexin V/PI apoptotic assay.

Given the in vitro and in vivo results, we can conclude that compound **3a** could be considered “hits” for further investigation and derivatization. In a posterior work, it will be fined tune-up and then will studied deeply to determine its mechanism of action and interaction with protein targets in human breast cancer cells.

## Figures and Tables

**Scheme 1:**
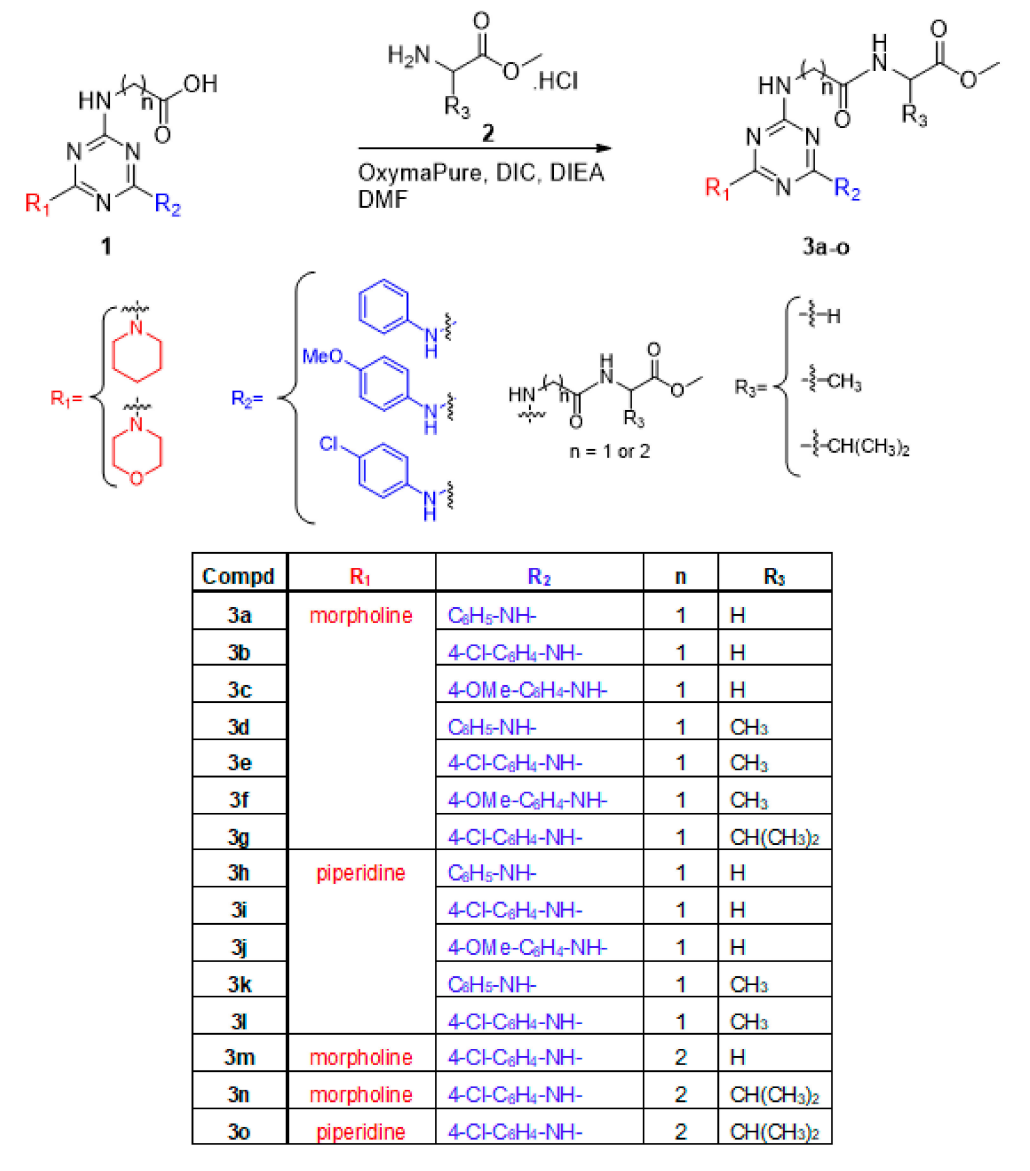
Synthesis of 4,6-disubstituted s-triazine dipeptide ester derivatives **3a–o**.

**Table 1:** Antiproliferative effects of **3a–o** in MCF-7, MDA-MB-231 and HCT-116 cells.

Compound	IC_50_ (μM)		
	MCF-7	MDA-MB-231	HCT-116
**3a**	0.82 ± 0.37	9.36 ± 1.15	17.89 ± 3.74
**3b**	6.18 ± 0.46	>50	>50
**3c**	13.50 ± 1.01	>50	>50
**3d**	3.57 ± 0.81	16.25 ± 0.63	>50
**3e**	4.35 ± 2.86	8.20 ± 1.68	>50
**3f**	8.02± 0.50	28.07 ±1.07	>50
**3g**	13.24 ± 0.60	24.13 ± 2.61	12.19 ± 1.74
**3h**	19.64 ± 0.08	>50	15.22 ± 2.04
**3i**	8.37 ± 0.77	22.26 ± 1.87	18.46 ± 1.45
**3j**	25.29 ± 2.75	>50	23.36 ± 3.43
**3k**	15.31 ± 2.49	13.26 ± 1.12	>50
**3l**	1.46 ± 0.69	8.31 ± 0.69	7.06 ± 0.27
**3m**	19.73 ± 1.48	26.66 ± 1.32	>50
**3n**	2.35 ± 1.06	8.48 ± 1.71	14.38 ± 1.37
**3o**	4.64 ± 1.58	15.71 ± 2.21	18.93 ± 2.82
**Tamoxifen**	5.12 ± 0.36	15.01± 0.12	26.41 ± 4.11

**Table 2:** Mortality response of zebrafish embryos to exposure to **3a–o**.

Compound	* LC_50_ (µM)	Compound	* LC_50_ (µM)
**3a**	NA	**3i**	6.22 ± 0.39
**3b**	NA	**3j**	80.15 ± 0.59
**3c**	NA	**3k**	5.98 ± 0.53
**3d**	NA	**3l**	4.25 ± 0.38
**3e**	2.37 ± 0.27	**3m**	5.58 ± 0.95
**3f**	28.36 ± 1.18	**3n**	5.84 ± 1.09
**3g**	132.14 ± 0.86	**3o**	1.97 ± 0.27
**3h**	10.08 ± 0.43	**Tamoxifen**	8.00 ± 0.95

* Mean of three biological replicates ± standard deviation. NA: no mortality was observed at or above a concentration of 1 mM.

## Supplementary Materials

The following are available online. Figures S1–S15: the ^1^H and ^13^C-NMR spectrum for the prepared compounds **3a–o**.

## Sample Availability

The data presented in this study are available in this article or supplementary material here.
