# Supplementary material for: The Antiproliferative and Apoptotic Effect of a Novel Synthesized S-Triazine Dipeptide Series, and Toxicity Screening in Zebrafish Embryos
Source: Molecules. 2021 Feb 22;26(4):1170. doi: 10.3390/molecules26041170 (PMC7926980; doi:10.3390/molecules26041170)

Supporting Information

The antiproliferative and apoptotic effect of novel synthesized *s*-triazine dipeptide series, and toxicity screening in zebrafish embryos

Azizah M. Malebari, Rakia Abd Alhameed, Zainab Almarhoon, Muhammad Farooq,* Mohammad A.M. Wadaan, Anamika Sharma and Beatriz G. de la Torre, Fernando Albericio,* Ayman El-Faham*

Table of content

Figure S1 ^{1}H-NMR and ^{13}C-NMR spectrum for 3a

Figure S2 ^{1}H-NMR and ^{13}C-NMR spectrum for 3b

Figure S3 ^{1}H-NMR and ^{13}C-NMR spectrum for 3c

Figure S4 ^{1}H-NMR and ^{13}C-NMR spectrum for 3a

Figure S5 ^{1}H-NMR and ^{13}C-NMR spectrum for 3e

Figure S6 ^{1}H-NMR and ^{13}C-NMR spectrum for 3f

Figure S7 ^{1}H-NMR and ^{13}C-NMR spectrum for 3a

Figure S8 ^{1}H-NMR and ^{13}C-NMR spectrum for 3h

Figure S9 ^{1}H-NMR and ^{13}C-NMR spectrum for 3i

Figure S10 ^{1}H-NMR and ^{13}C-NMR spectrum for 3j

Figure S11 ^{1}H-NMR and ^{13}C-NMR spectrum for 3k

Figure S12 ^{1}H-NMR and ^{13}C-NMR spectrum for 3l

Figure S13 ^{1}H-NMR and ^{13}C-NMR spectrum for 3m

Figure S14 ^{1}H-NMR and ^{13}C-NMR spectrum for 3n

Figure S15 ^{1}H-NMR and ^{13}C-NMR spectrum for 3o

Figure S1: ^{1}H-NMR and ^{13}C-NMR spectrum for 3a

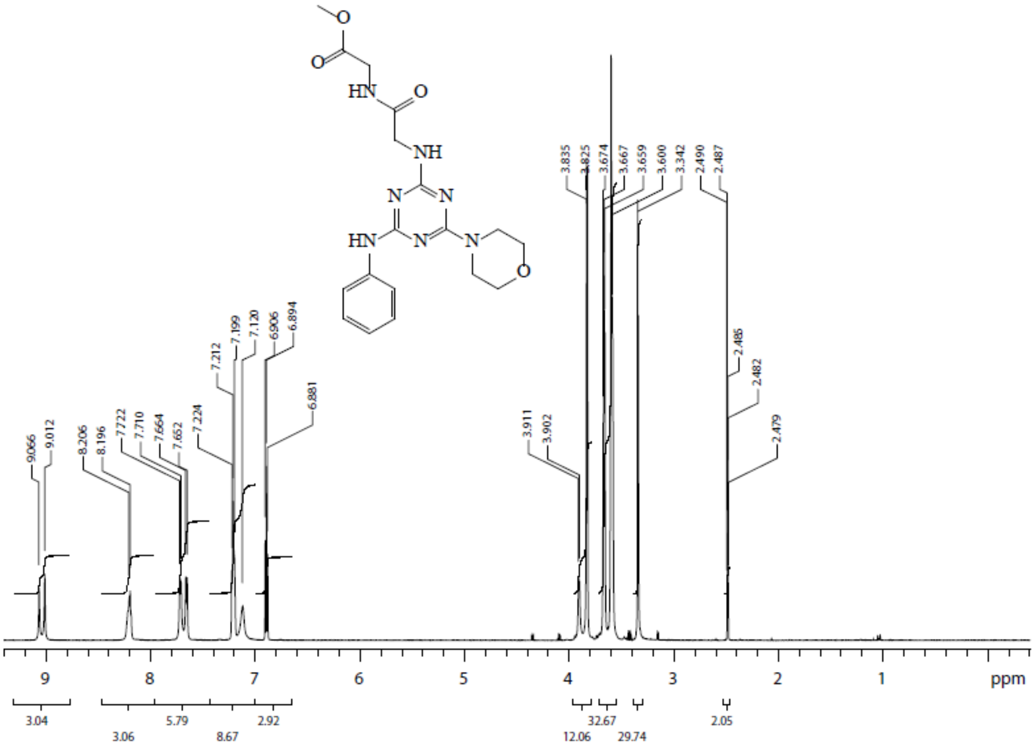

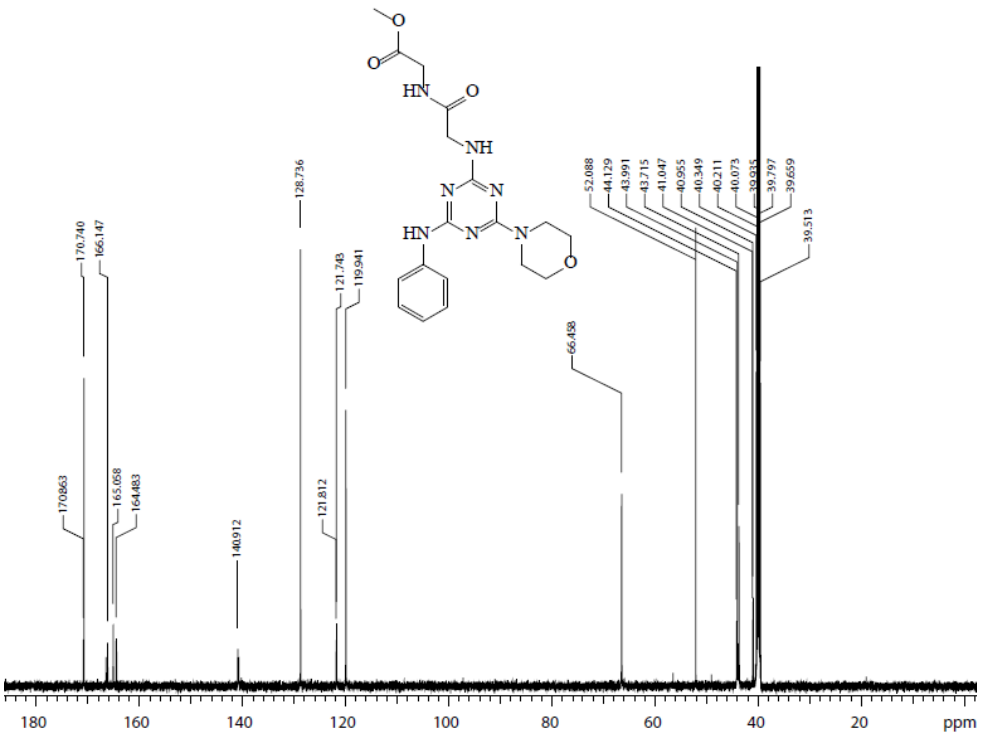

Figure S2: ^{1}H-NMR and ^{13}C-NMR spectrum for 3b

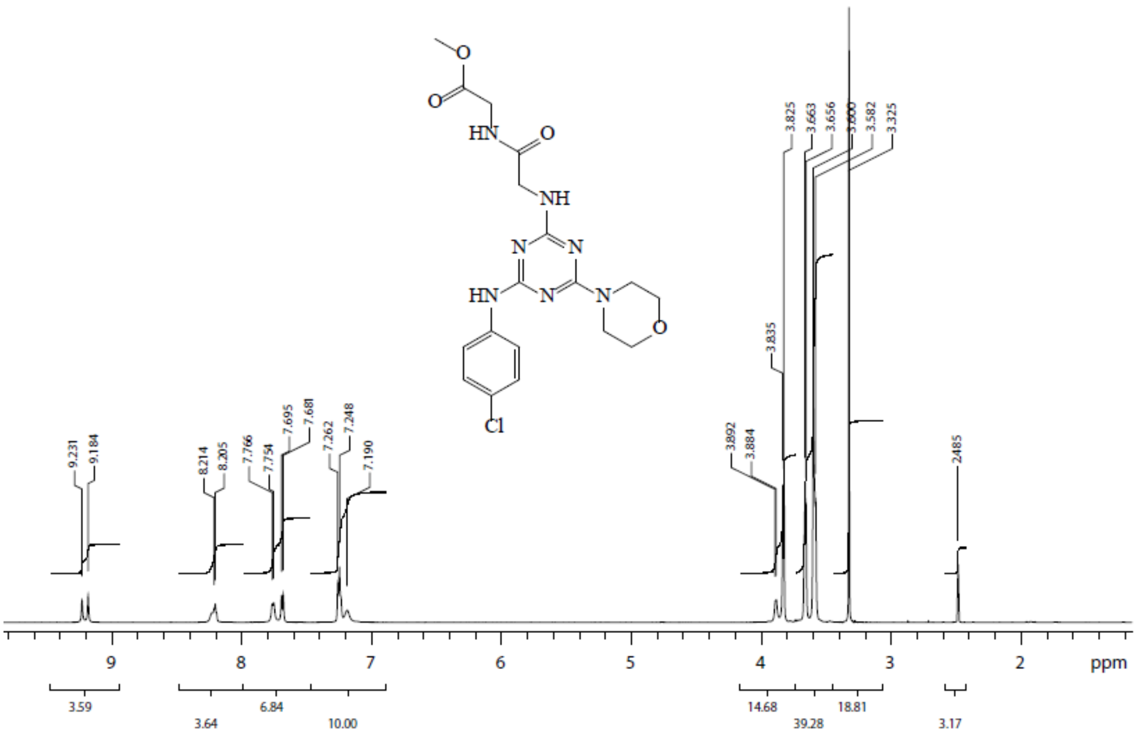

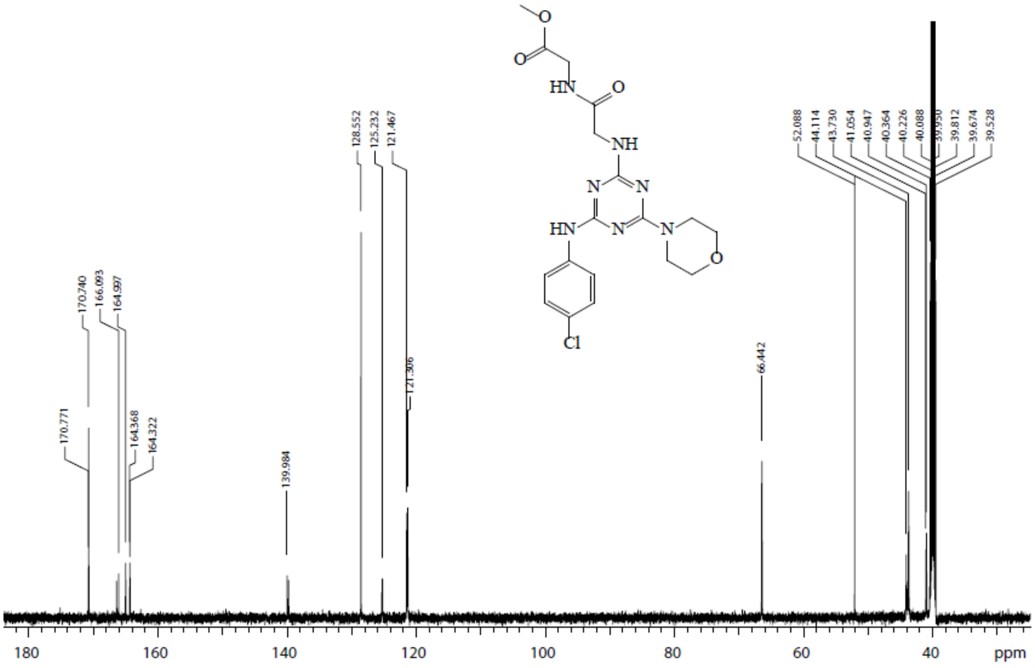

Figure S3: ^{1}H-NMR and ^{13}C-NMR spectrum for 3c

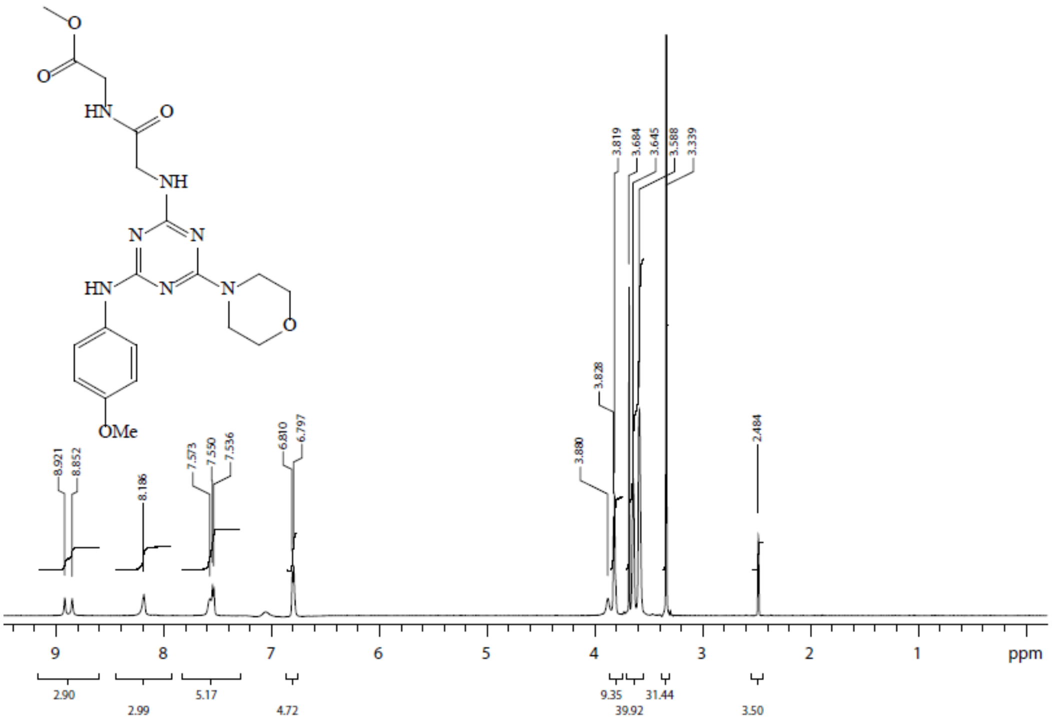

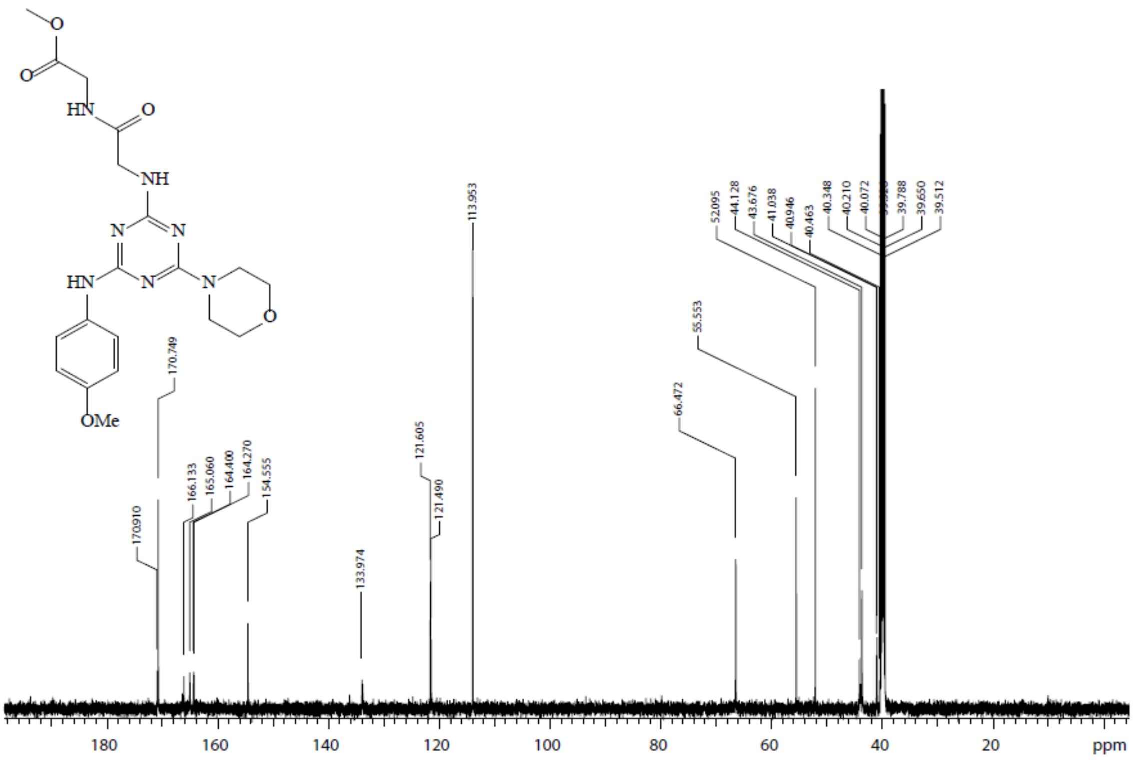

Figure S4: ^{1}H-NMR and ^{13}C-NMR spectrum for 3a

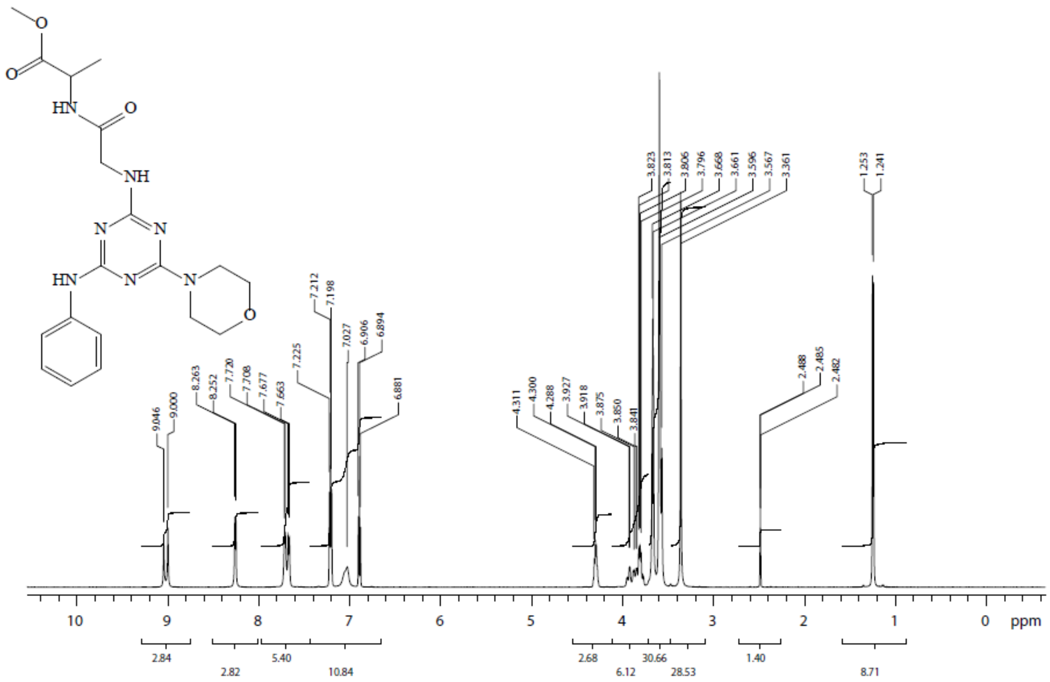

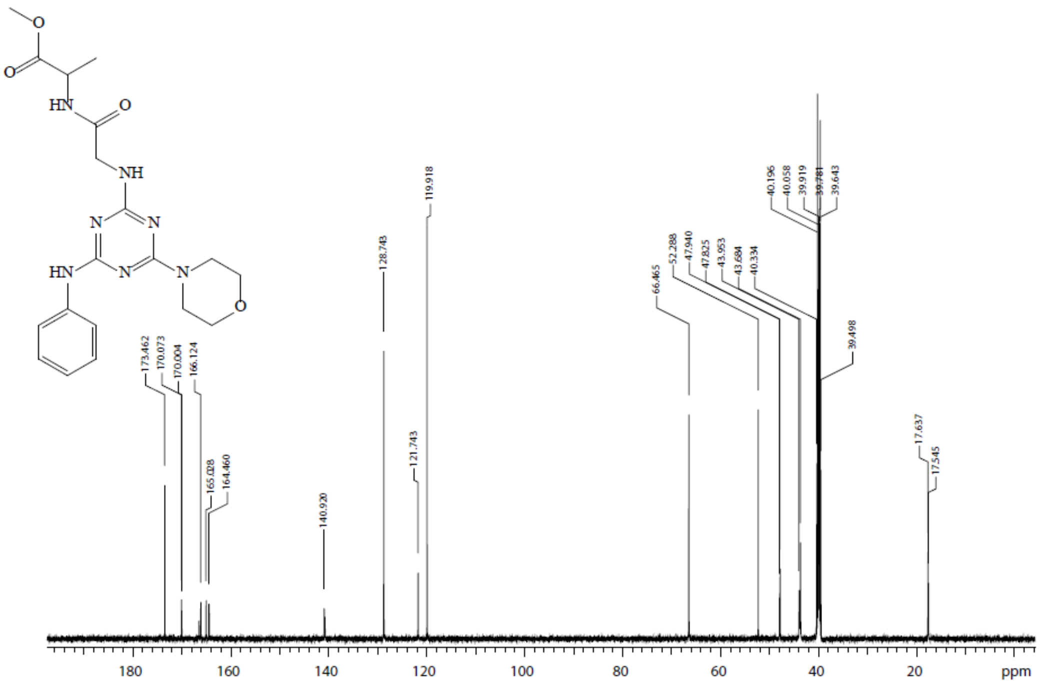

Figure S5: ^{1}H-NMR and ^{13}C-NMR spectrum for 3e

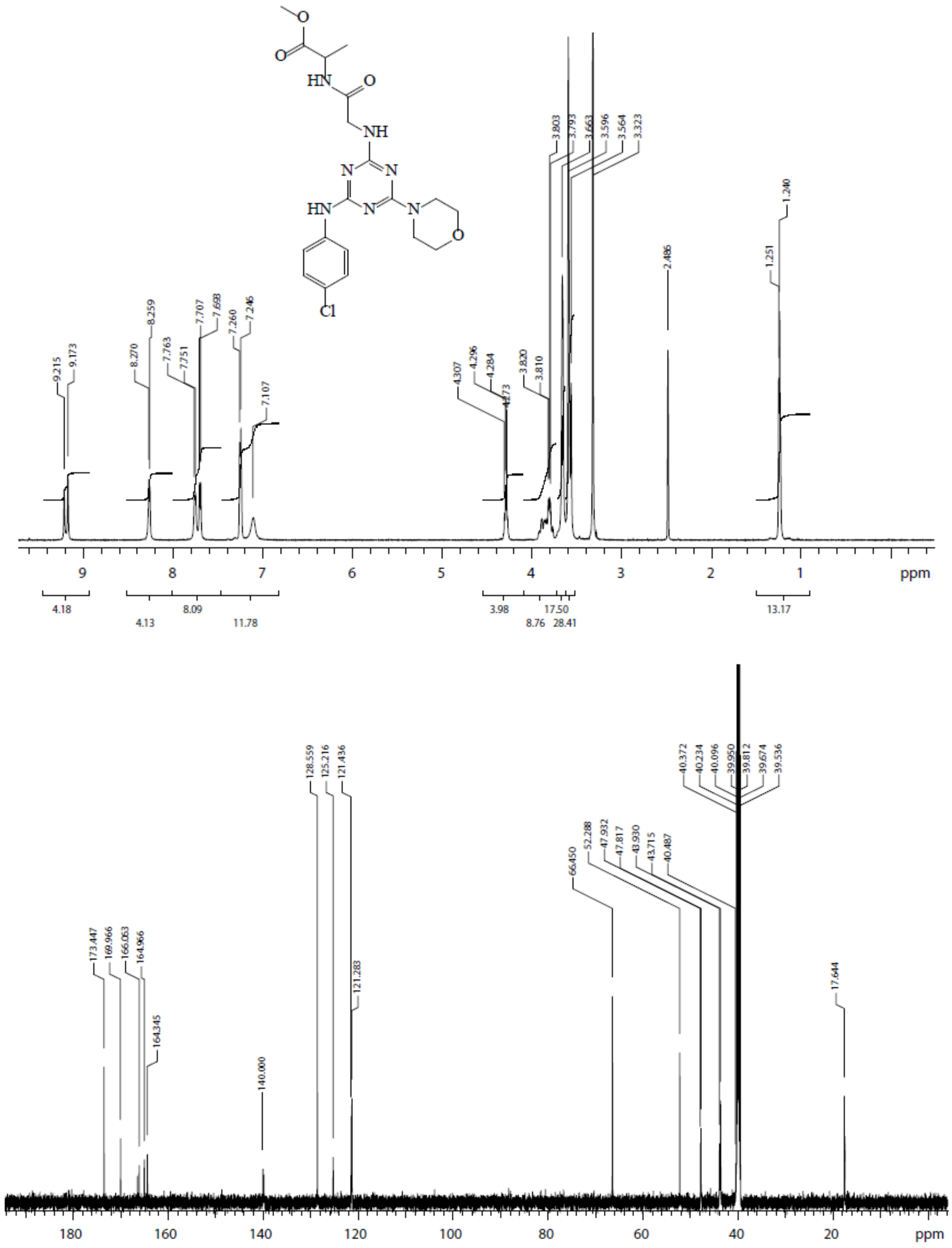

Figure S6: ^{1}H-NMR and ^{13}C-NMR spectrum for 3f

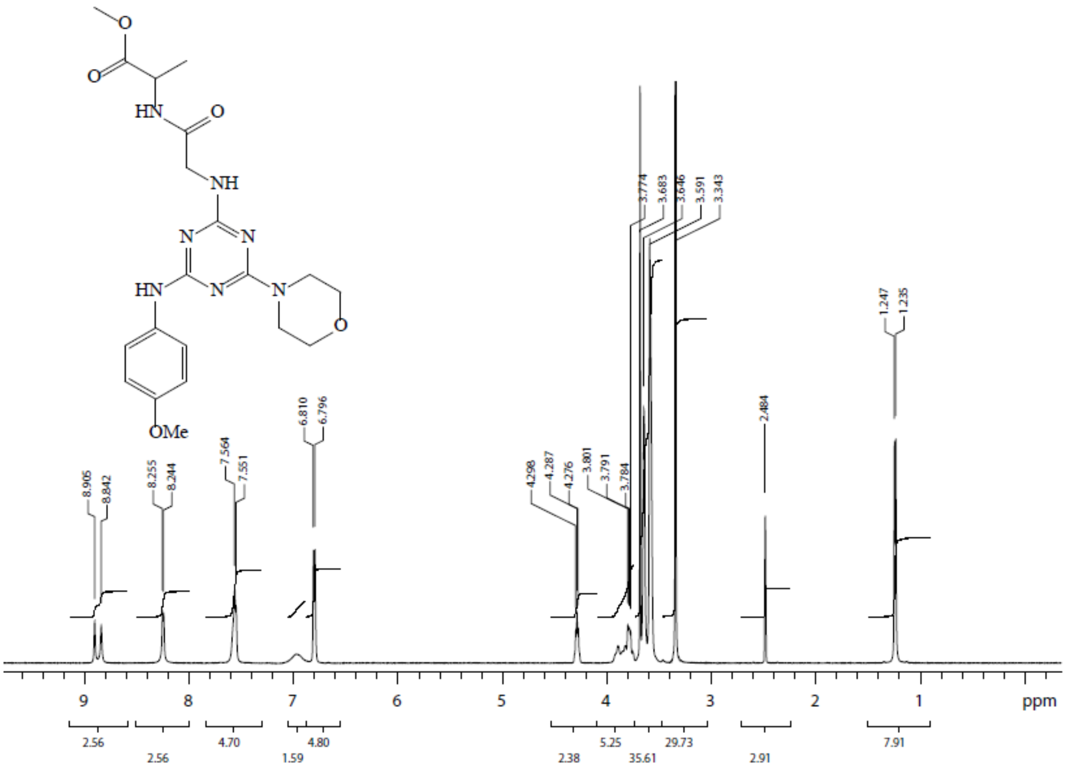

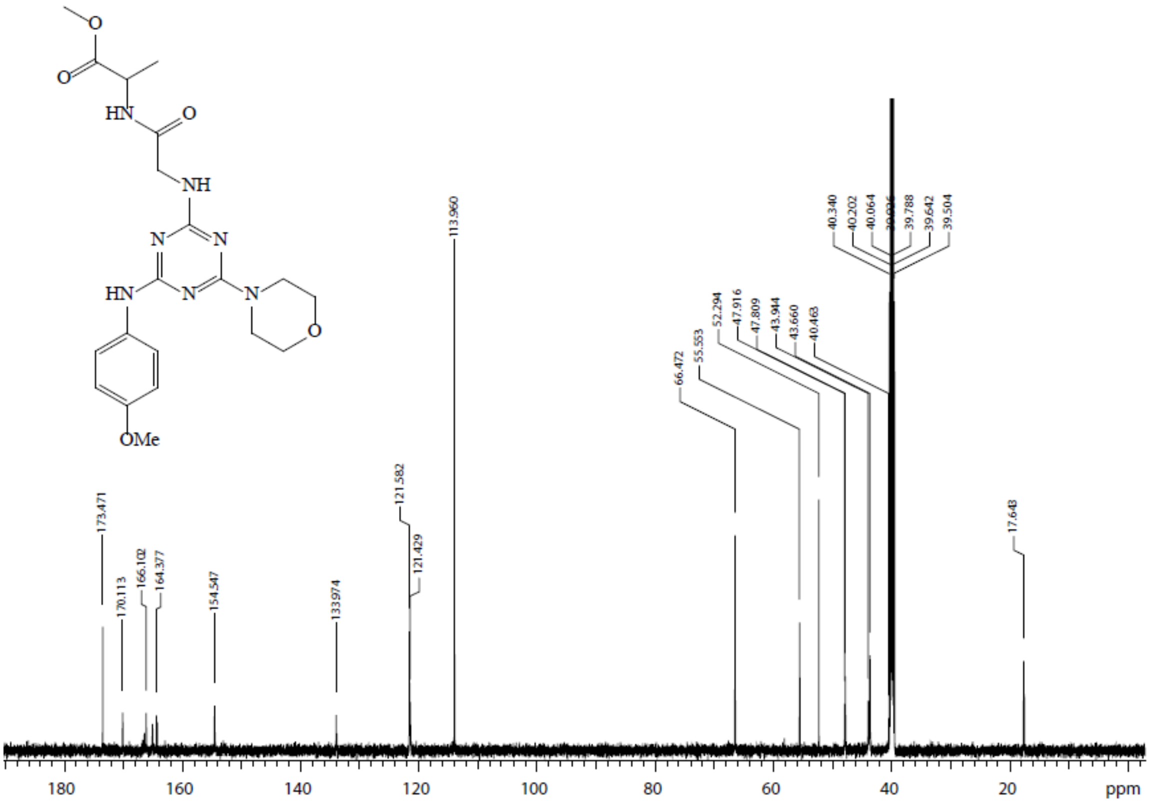

Figure S7: ^{1}H-NMR and ^{13}C-NMR spectrum for 3a

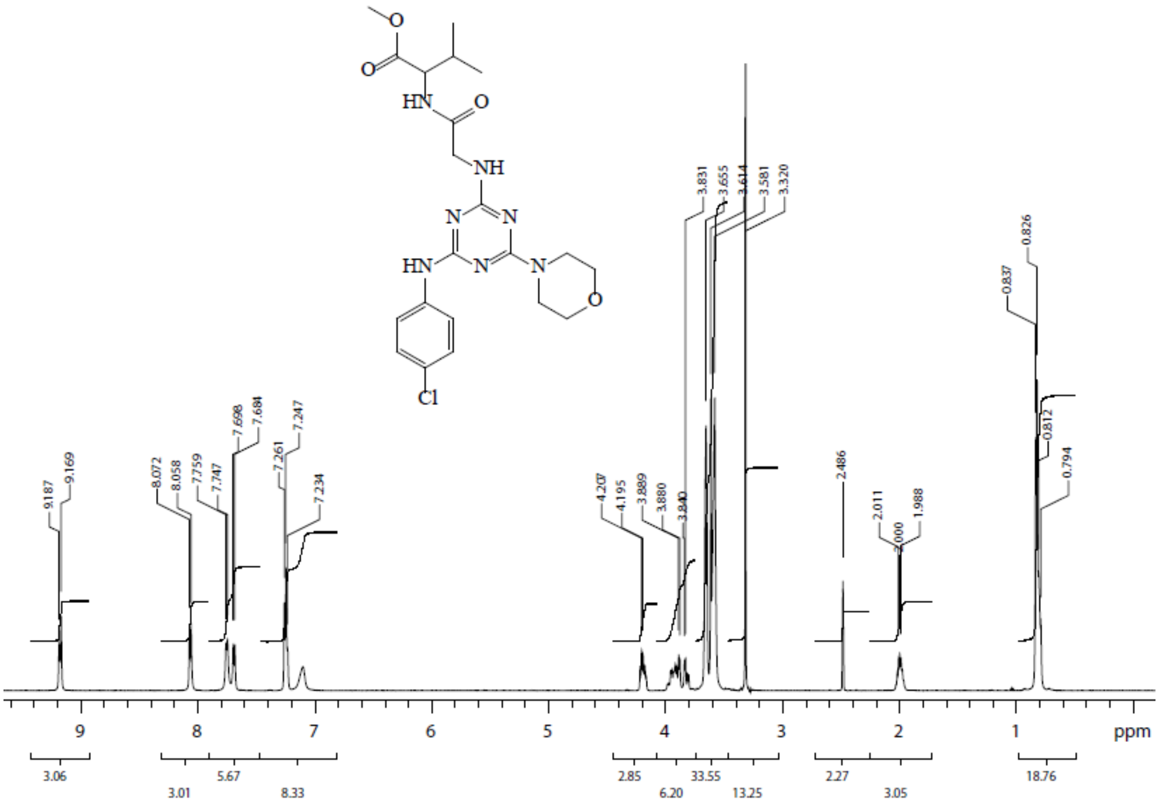

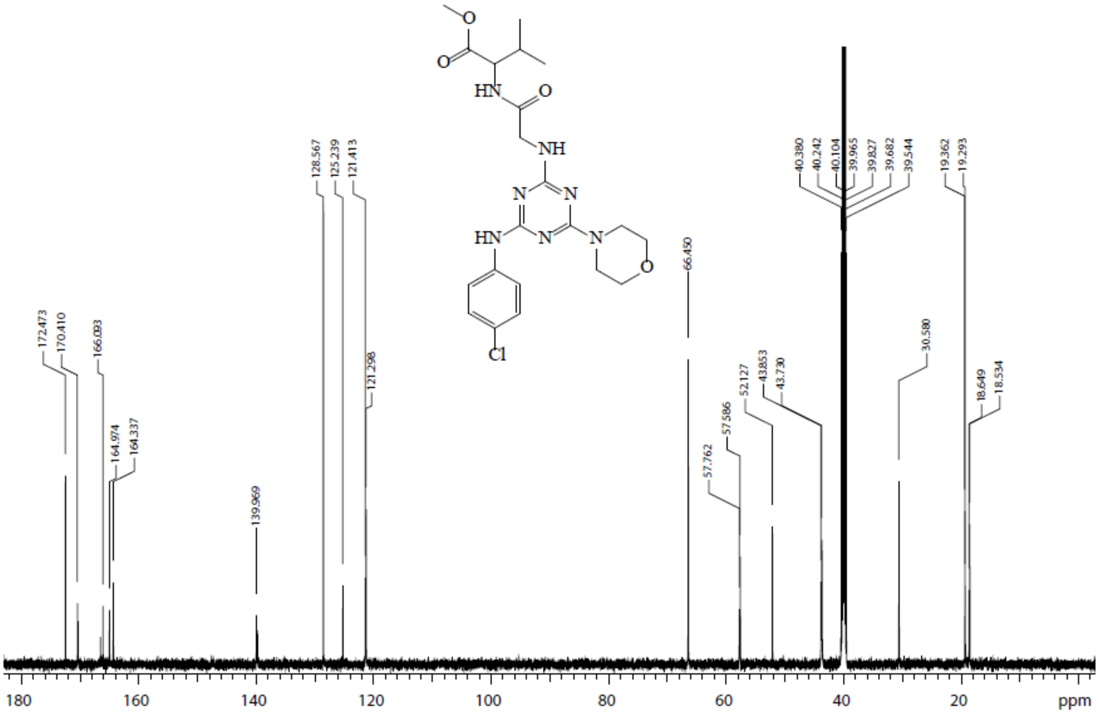

Figure S8: ^{1}H-NMR and ^{13}C-NMR spectrum for 3h

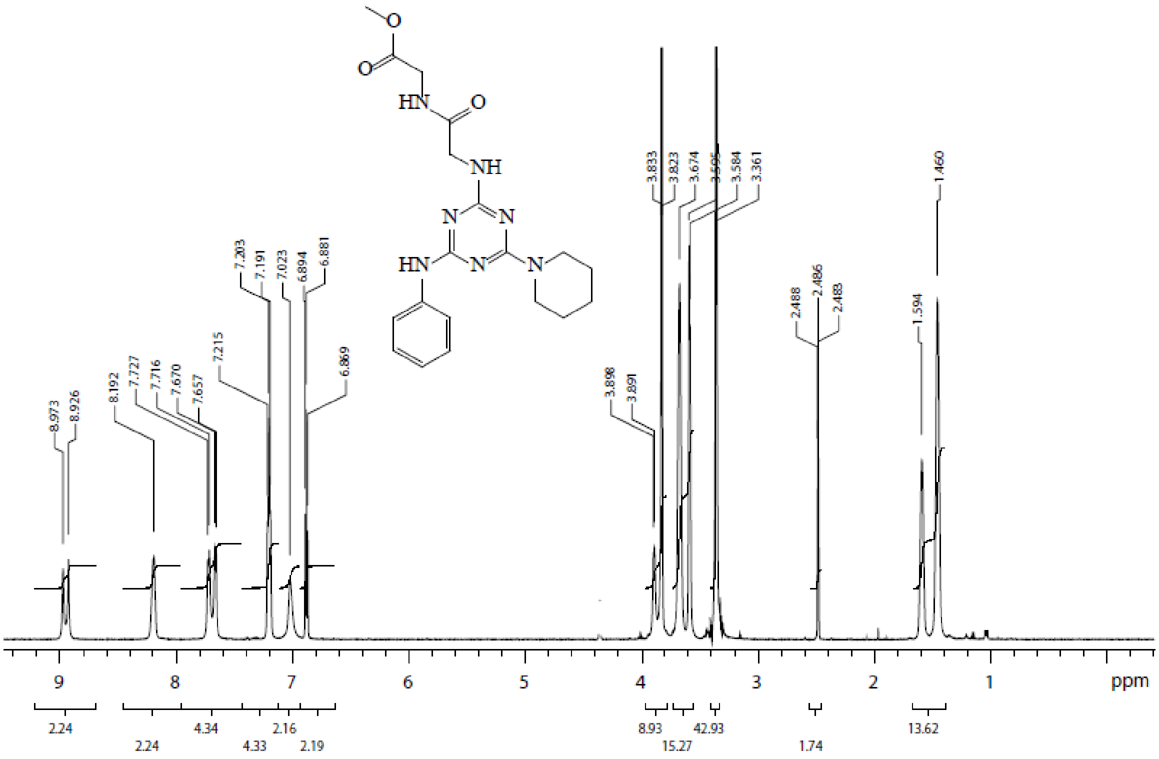

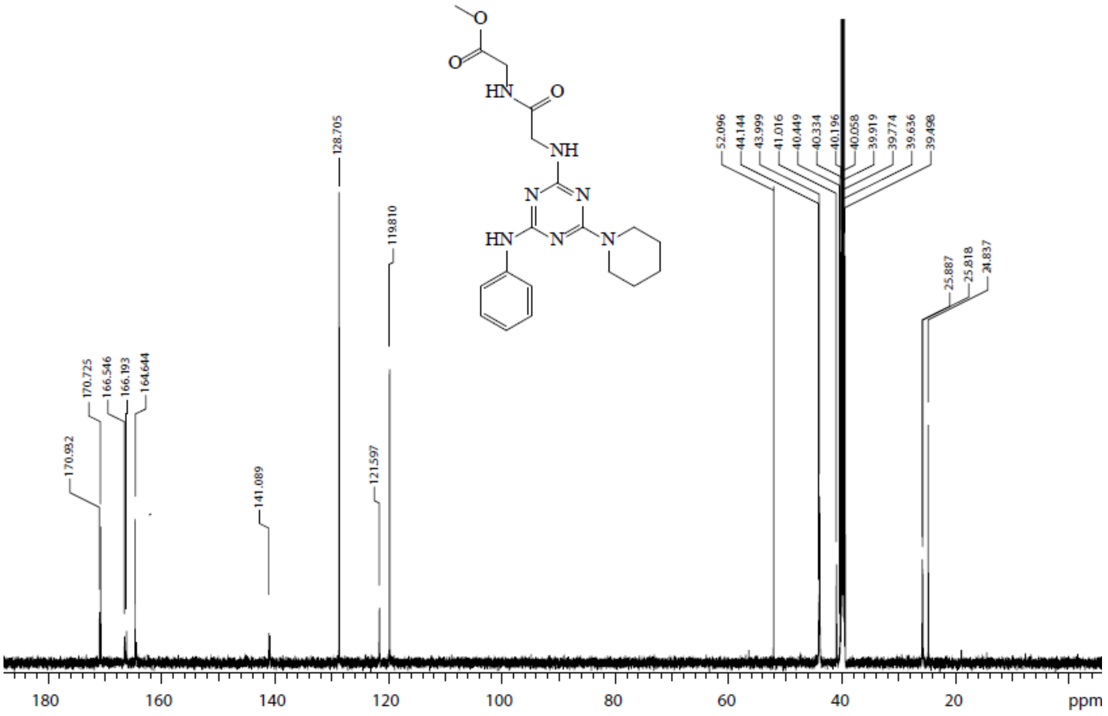

Figure S9: ^{1}H-NMR and ^{13}C-NMR spectrum for 3i

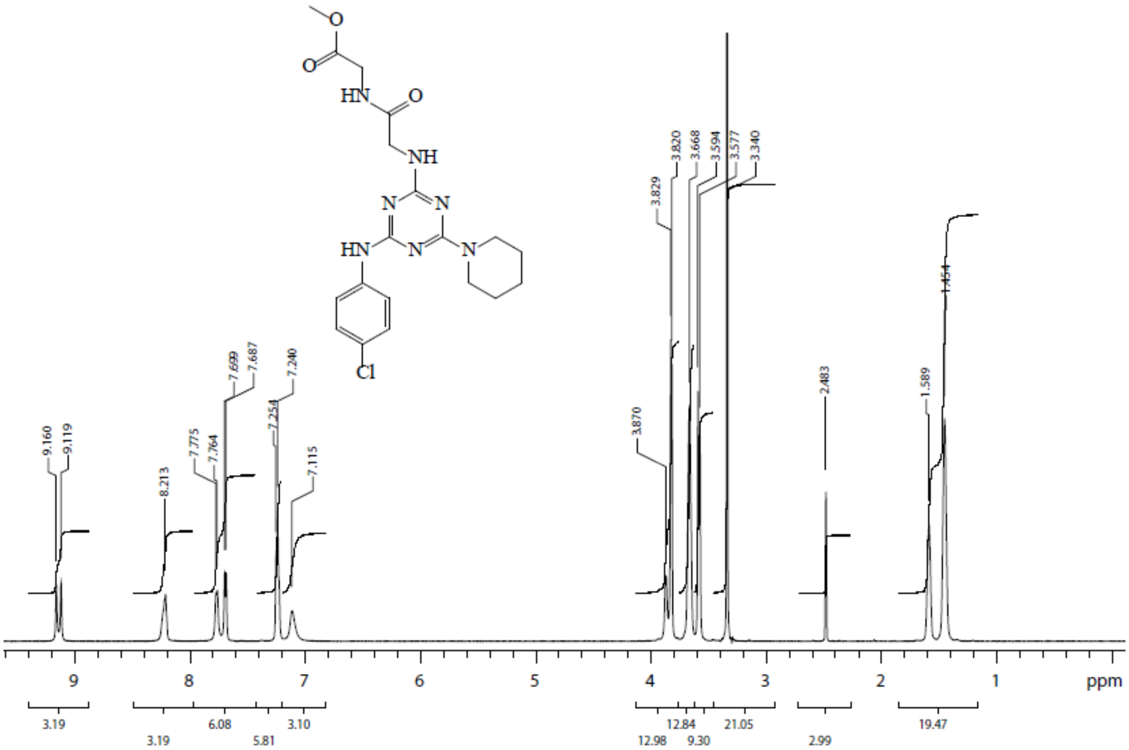

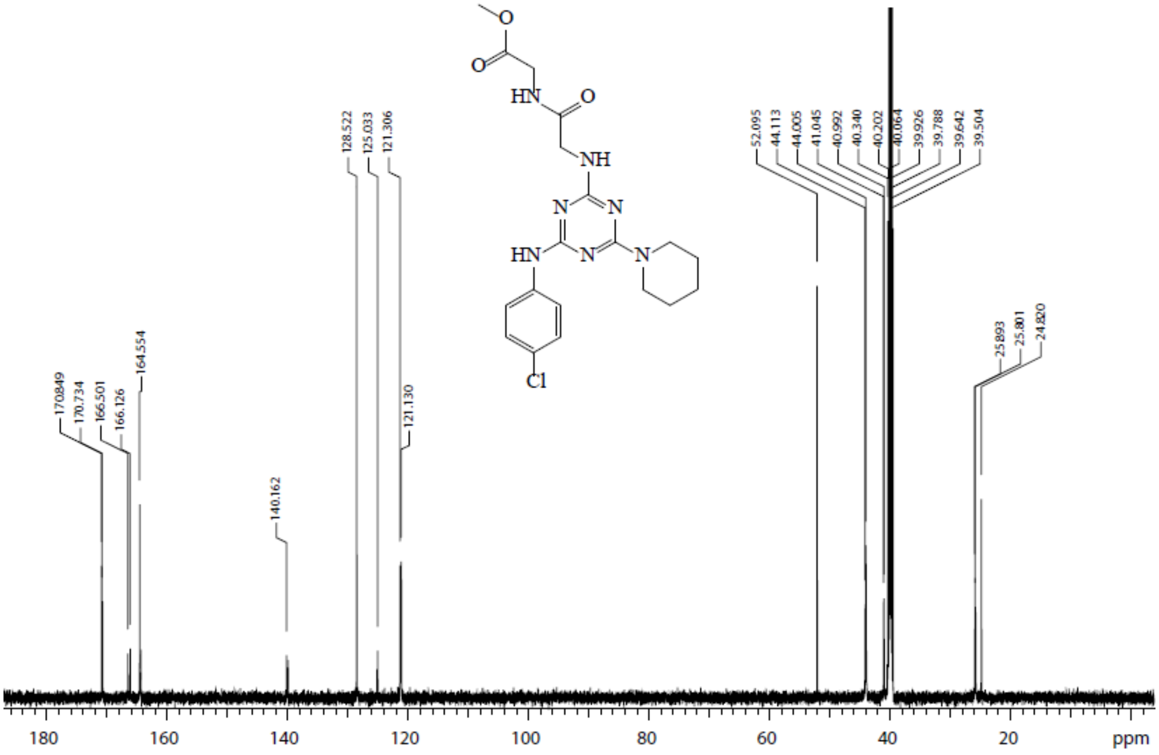

Figure S10: ^{1}H-NMR and ^{13}C-NMR spectrum for 3j

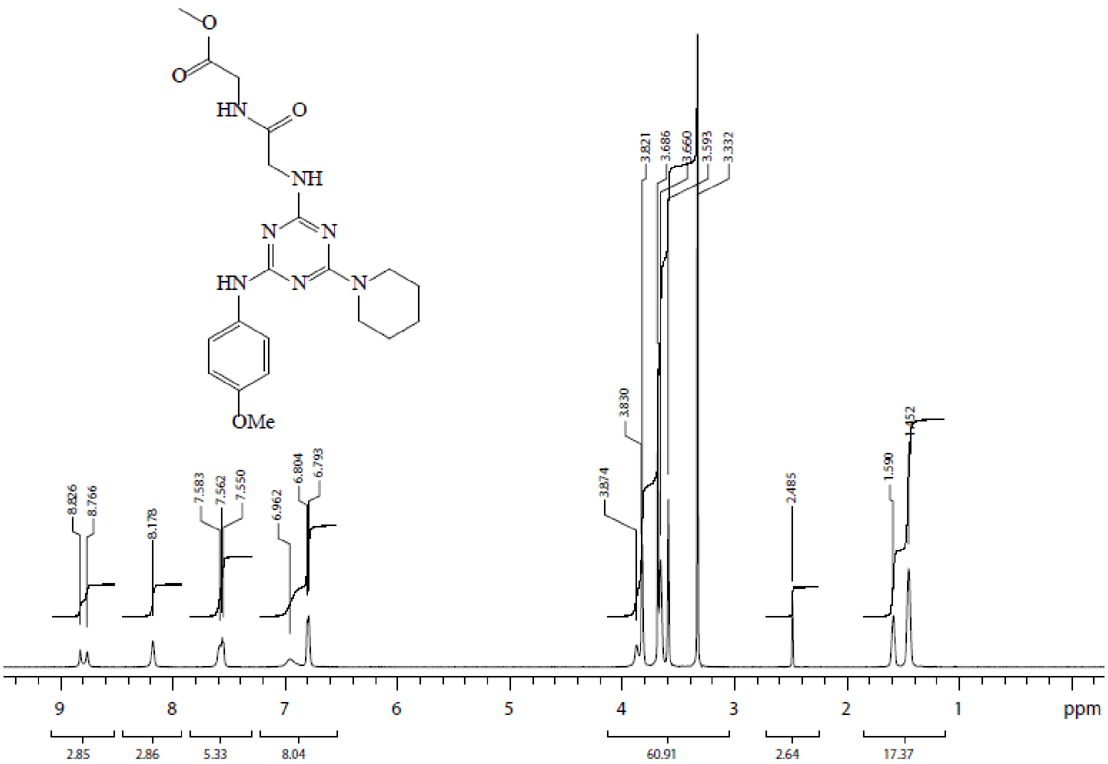

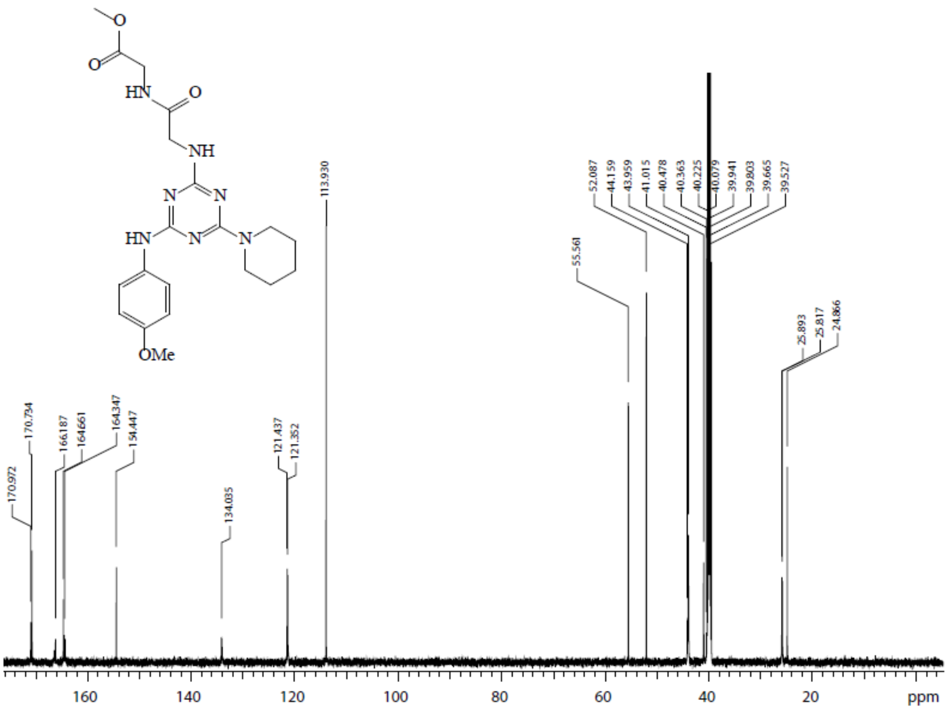

Figure S11: ¹H-NMR and ¹³C-NMR spectrum for 3k

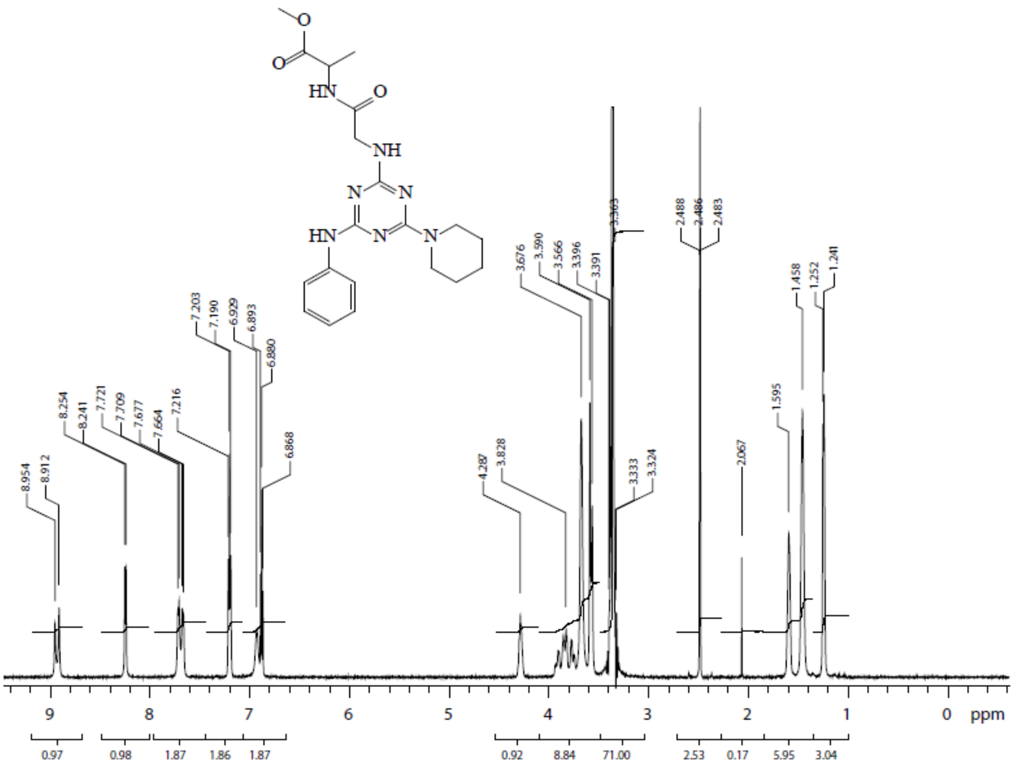

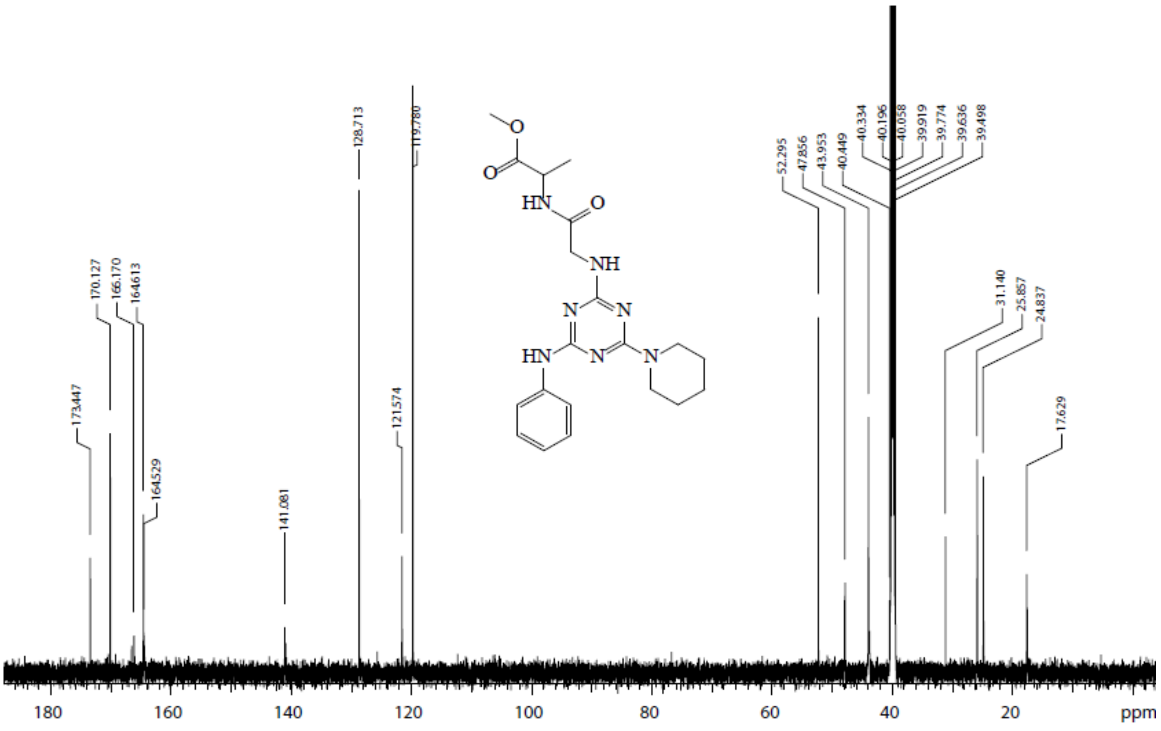

Figure S12: ^{1}H-NMR and ^{13}C-NMR spectrum for 3l

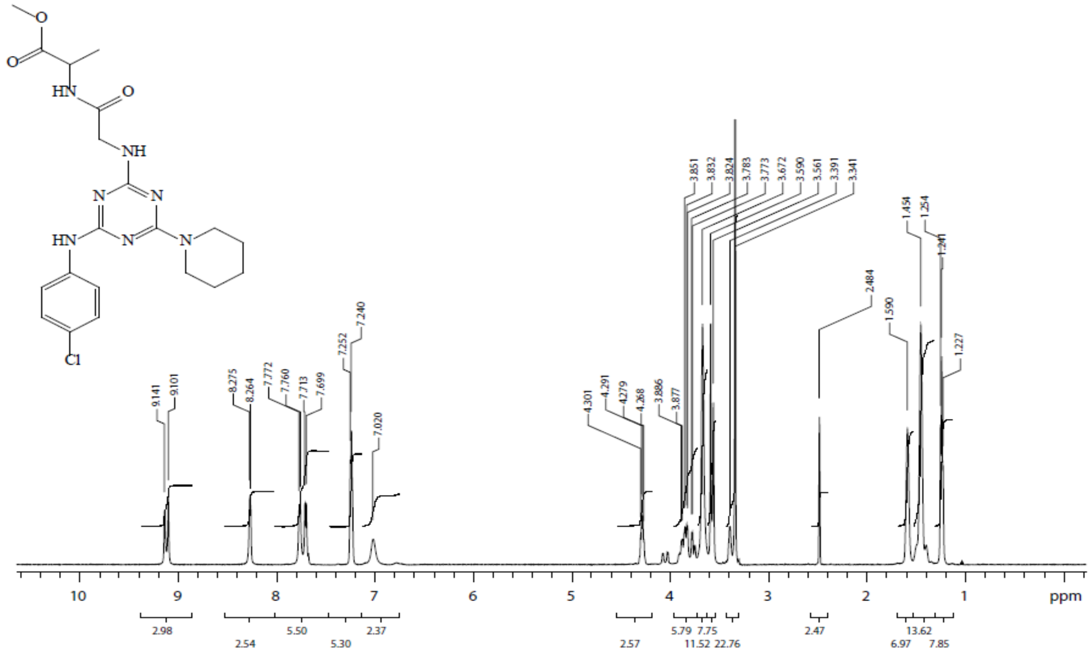

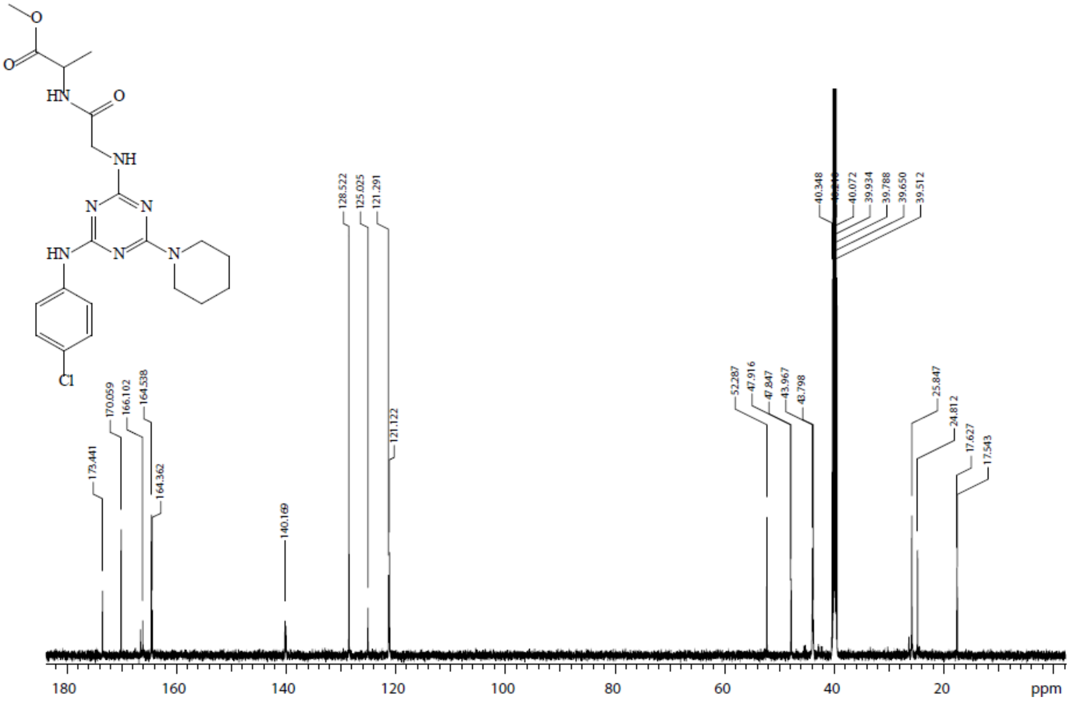

Figure S13: ^{1}H-NMR and ^{13}C-NMR spectrum for 3m

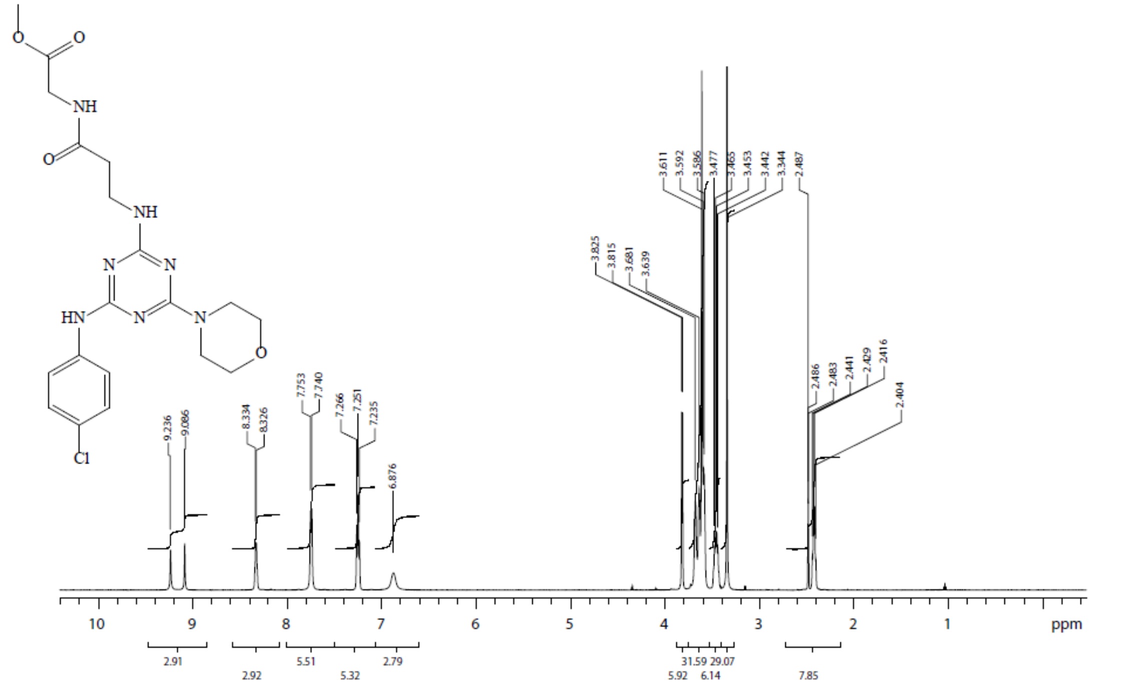

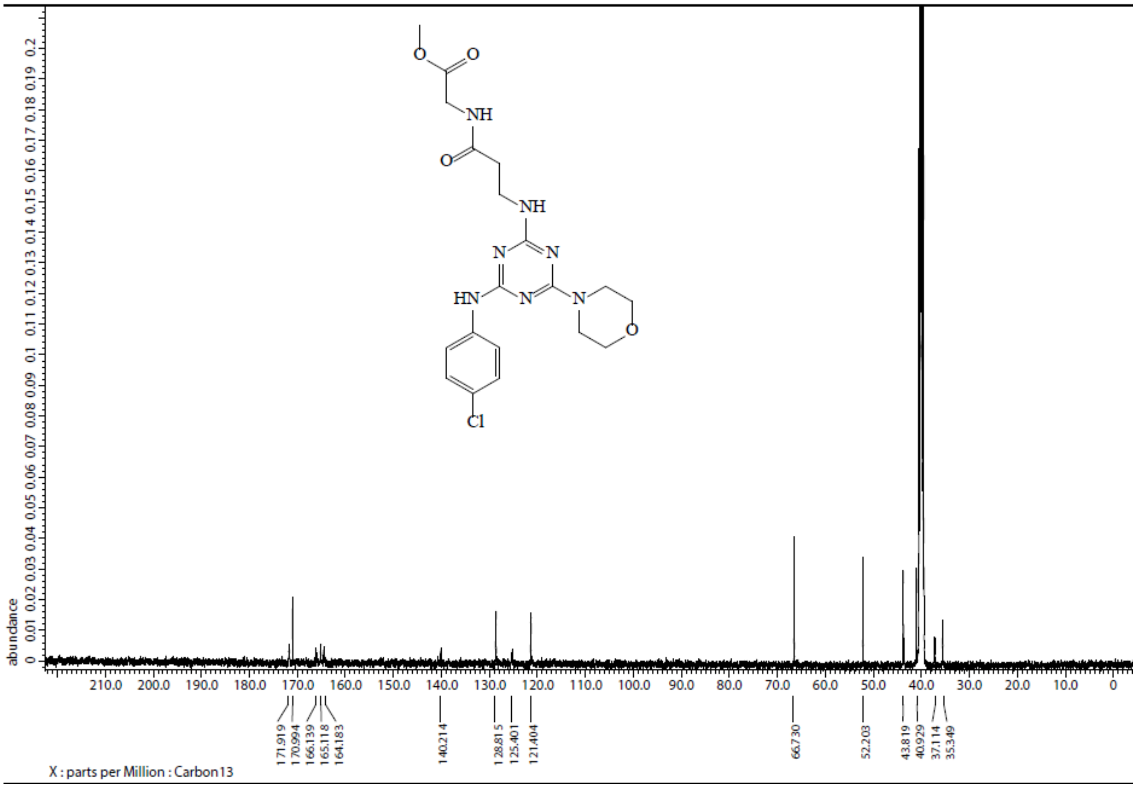

Figure S14: ^{1}H-NMR and ^{13}C-NMR spectrum for 3n

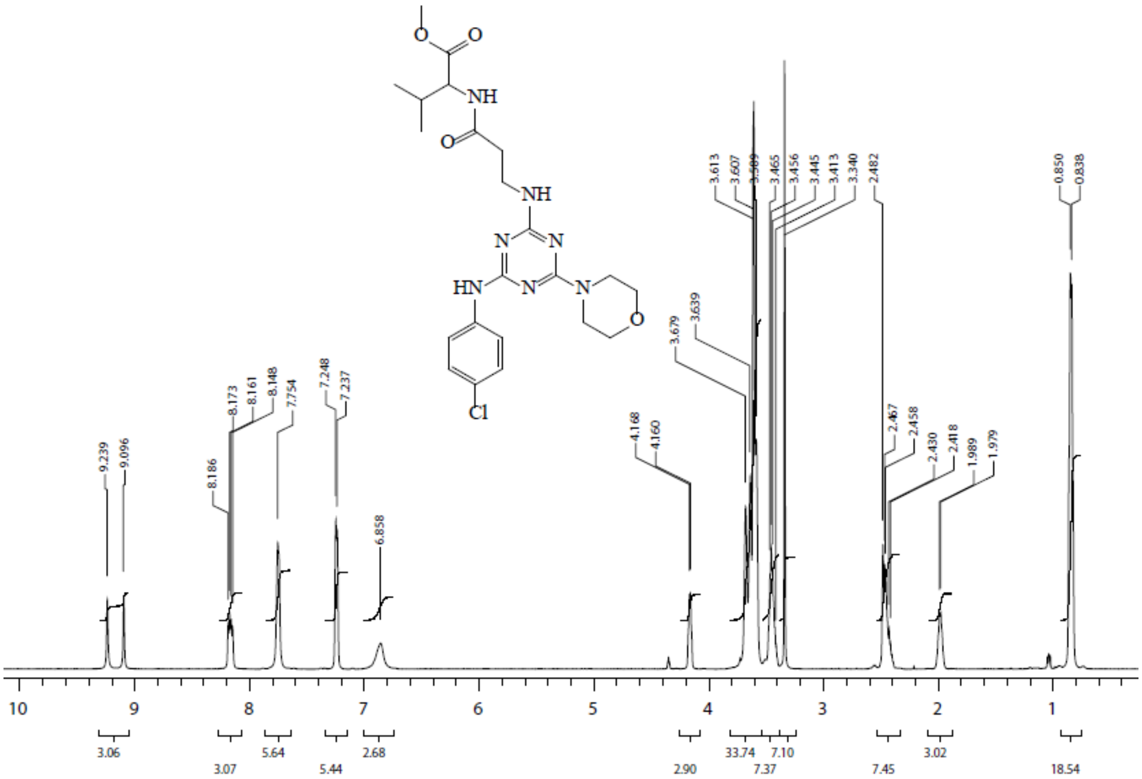

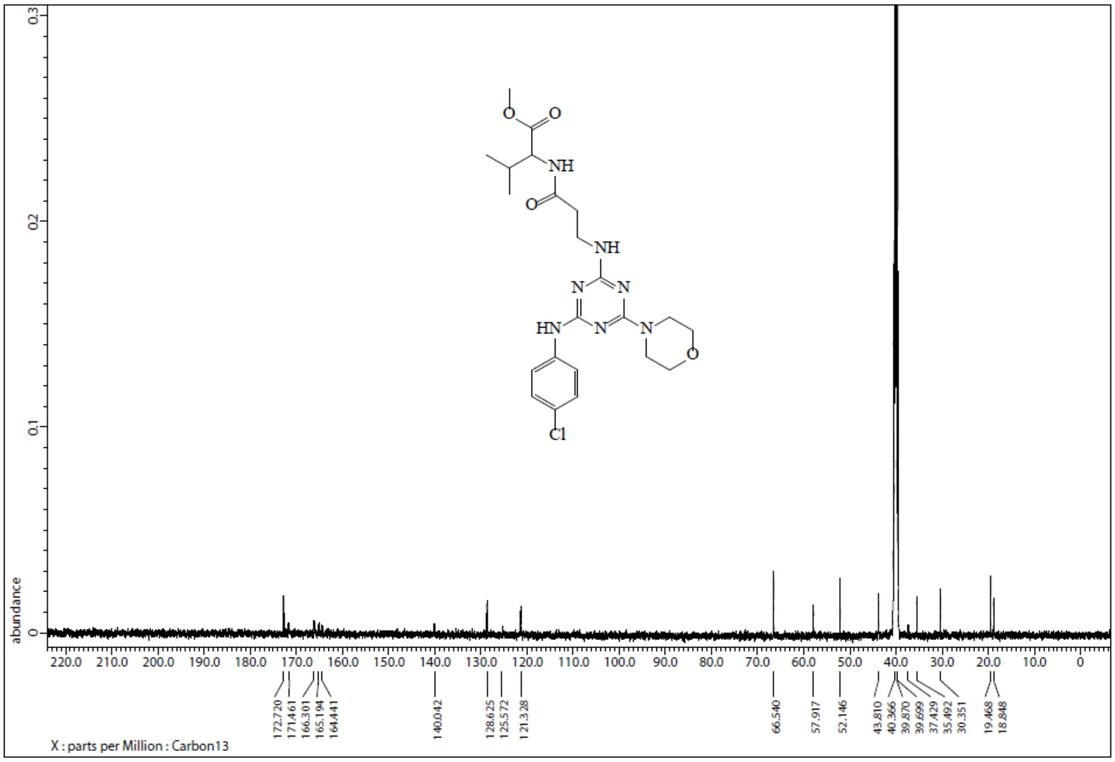

Figure S15: ^{1}H-NMR and ^{13}C-NMR spectrum for 3o

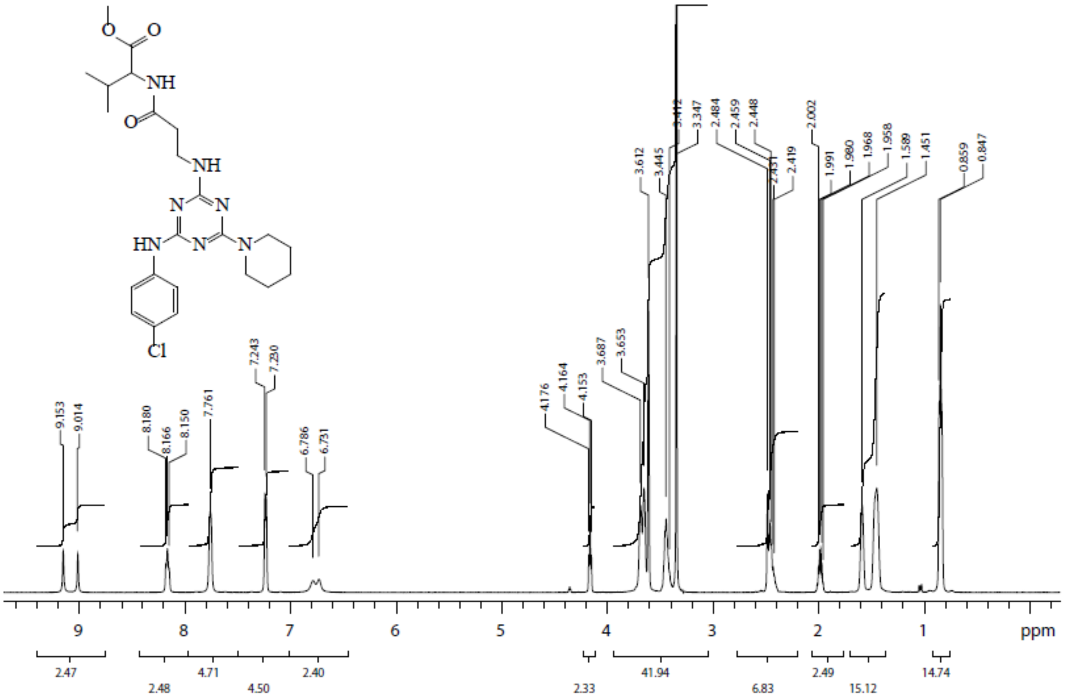

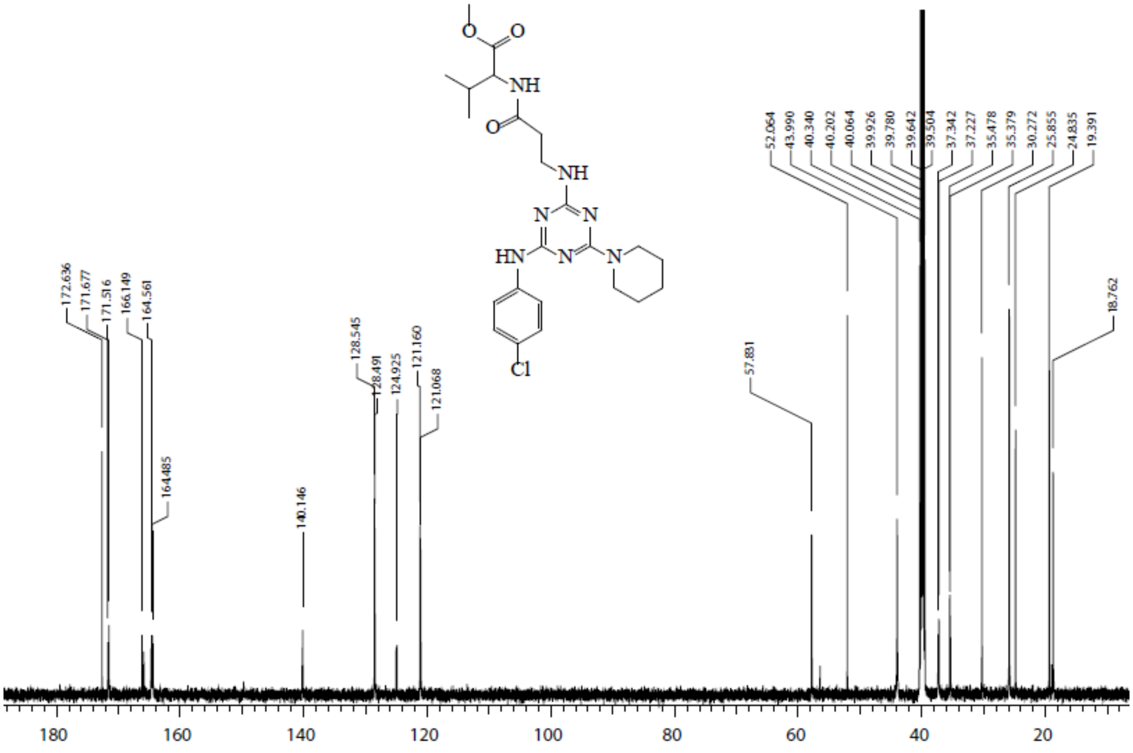

Supplement: Supplementary file 1 [file molecules-26-01170-s001.pdf]
